# Convergence Rates of Forward–Douglas–Rachford Splitting Method

**DOI:** 10.1007/s10957-019-01524-9

**Published:** 2019-04-11

**Authors:** Cesare Molinari, Jingwei Liang, Jalal Fadili

**Affiliations:** 1 GREYC, ENSICAEN, CNRS, Normandie Université, Caen, France; 2 DAMTP, University of Cambridge, Cambridge, UK

**Keywords:** Forward–Douglas–Rachford, Forward–Backward, Bregman distance, Partial smoothness, Finite identification, Local linear convergence, 49J52, 65K05, 65K10, 90C25

## Abstract

Over the past decades, operator splitting methods have become ubiquitous for non-smooth optimization owing to their simplicity and efficiency. In this paper, we consider the Forward–Douglas–Rachford splitting method and study both global and local convergence rates of this method. For the global rate, we establish a sublinear convergence rate in terms of a Bregman divergence suitably designed for the objective function. Moreover, when specializing to the Forward–Backward splitting, we prove a stronger convergence rate result for the objective function value. Then locally, based on the assumption that the non-smooth part of the optimization problem is partly smooth, we establish local linear convergence of the method. More precisely, we show that the sequence generated by Forward–Douglas–Rachford first (i) identifies a smooth manifold in a finite number of iteration and then (ii) enters a local linear convergence regime, which is for instance characterized in terms of the structure of the underlying active smooth manifold. To exemplify the usefulness of the obtained result, we consider several concrete numerical experiments arising from applicative fields including, for instance, signal/image processing, inverse problems and machine learning.

## Introduction

Operator splitting methods are iterative schemes to solve inclusion and optimization problems by decoupling the original problem into subproblems that are easy to solve. These schemes evaluate the individual operators, their resolvents, the linear operators, all separately at various points in the course of iteration, but never the resolvents of sums nor of composition by a linear operator. Since the first operator splitting method developed in the 1970s for solving structured monotone inclusion problems, the class of splitting methods has been regularly enriched with increasingly sophisticated algorithms as the structure of problems to handle become more complex. We refer the readers to [1] and references therein for a through account of operator splitting methods.

In this paper, we consider a subspace constrained optimization problem, where the objective function is the sum of a proper convex and lower semi-continuous function and a convex smooth differentiable function with Lipschitz gradient. To efficiently handle the constraint, a provably convergent algorithm is Forward–Douglas–Rachford splitting algorithm (FDR) [2], which is a hybridization of Douglas–Rachford splitting algorithm (DR) [3] and Forward–Backward splitting algorithm (FB) [4]. FDR is also closely related to the generalized Forward–Backward splitting algorithm (GFB) [5, 6] and the three-operator splitting method (TOS) [7].

Global sublinear convergence rate to asymptotic regularity of the sequence generated by FDR (hence all the above-mentioned algorithms) has been recently established in the literature, from the perspective of Krasnosel’skiĭ–Mann fixed-point iteration; see, for instance, [8] and the references therein. This allows to exhibit convergence rates of the distance of 0 to the objective subdifferential evaluated at the iterate. However, very limited results have been reported in the literature on the convergence rate of the objective function value for FDR, except for certain specific cases. For instance, the objective convergence rate of Forward–Backward splitting and its accelerated versions are now well understood [9–14]. These results rely essentially on some monotonicity property of a properly designed Lyapunov function. Given that FDR is fixed-point algorithm, it is much more difficult or even impossible to study the convergence rate of the objective function value. Indeed, these algorithms generate several different points along the course of iteration, making it rather challenging to design a proper Lyapunov function (as we shall see for the FDR algorithm in Sect. 4).

Recently, local linear convergence of operator splitting algorithms for optimization has recently attracted a lot of attention; see [15] for Forward–Backward-type methods, [16] for Douglas–Rachford splitting, and [17] for Primal–Dual splitting algorithms. This work particularly exploits the underlying geometric structure of the optimization problems, achieving a local linear convergence result without assuming conditions like strong convexity, unlike what is proved in [8, 18]. In practice, local linear convergence of FDR algorithm is also observed. However, to our knowledge, there is no theoretical explanation available for this local behaviour.

*Main Contributions* In this paper, we study both the global and local convergence rates of the FDR algorithm. Our main contributions consist of both global and local aspects. First, the global convergence behaviour is studied under a general real Hilbert space setting.

- In Sect. 4, we first prove the convergence of the newly proposed non-stationary FDR scheme (6). This is achieved by capturing non-stationarity as an error term. The proof exploits a general result on inexact and non-stationary Krasnosel’skiĭ–Mann  fixed-point iteration developed in [8].
- We design a Bregman divergence as a meaningful convergence criterion. Under the standard assumptions, we show pointwise and ergodic convergence rates of this criterion (Theorem 4.2). When specializing the result to Forward–Backward splitting, we obtain a stronger claim for the objective convergence rate of the method. The allowed range of step size for the latter rate to hold is twice larger than the one known in the literature.

For local convergence analysis, we turn to finite dimension as partial smoothness, which is at the heart of this part is only available in the Euclidean setting.

- Finite Time Activity Identification Under the assumption that the non-smooth component of the optimization problem is partly smooth around a global minimizer relative to its smooth submanifold (see Definition 2.4) and under a non-degeneracy condition (see (31)), we show in Sect. 5 (Theorem 5.1) that the sequence generated by the non-stationary FDR identifies in finite time the solution submanifold. In plain words, this means that, after a finite number of iterations, the sequence enters the submanifold and never leaves it. We also provide a bound on the number of iterations to achieve identification.
- Local Linear Convergence Exploiting the finite identification property, we then show that the sequence generated by non-stationary FDR converges locally linearly. We characterize the convergence rate precisely based on the properties of the identified partial smoothness submanifolds.
- Three-operator Splitting Given the close relation between the three-operator splitting method and FDR, in Sect. 5.4, we extend the above local linear convergence result to the case of the three-operator splitting algorithm.

*Relation to Prior Work* The convergence rate of the objective value for FDR has been studied in [18]. There, the author presented ergodic and pointwise convergence rates on the objective value under different (more or less stringent) assumptions imposed on the non-smooth function in the objective (1). Without any further assumptions other than (A.1)–(A.5), the author proved a pointwise convergence rate on a criterion associated to the objective value, but in absolute value (see [18, Theorem 3.5]). However, this rate seems quite pessimistic. (It suggests that FDR is as slow as subgradient descent.) Moreover, there is no non-negativity guarantee for such criterion and the obtained rate is thus of a quite limited interest. Improving this rate on the objective value requires quite strong assumptions on the non-smooth component.

As far as local linear convergence of the sequence in the absence of strong convexity is concerned, it has received an increasing attention in the past few years in the context of first-order proximal splitting methods. The key idea here is to exploit the geometry of the underlying objective around its minimizers. This has been done for instance in [15–17, 19] for the FB scheme, Douglas–Rachford splitting/ADMM and Primal–Dual splitting, under the umbrella of partial smoothness. The error bound property,^1^ as highlighted in the seminal work of [22, 23], is used by several authors to study linear convergence of first-order descent-type algorithms, and in particular FB splitting; see, for example, [20, 21, 24, 25]. However, to the best of our knowledge, we are not aware of local linear convergence results for the FDR algorithm.

*Paper Organization* The rest of the paper is organized as follows. In Sect. 2, we recall some classical material on convex analysis and operator theory that are essential to our exposition. We then introduce the notion of partial smoothness. The problem statement and FDR algorithm are presented in Sect. 3. The global convergence analysis is presented in Sect. 4, followed by finite identification and local convergence analysis in Sect. 5. Several numerical experiments are presented in Sect. 6. Some introductory material on smooth Riemannian manifolds is gathered in “Appendix”.

## Preliminaries

Throughout the paper, $\mathcal {H}$ is a Hilbert space equipped with scalar product $\langle \cdot ,\,\cdot \rangle$ and norm ${\Vert } \cdot {\Vert }$. $\text {Id}$ denotes the identity operator on $\mathcal {H}$. $\Gamma _0(\mathcal {H})$ denotes the set of proper convex and lower semi-continuous functions on $\mathcal {H}$.

*Sets* For a non-empty convex set $C \subset \mathcal {H}$, $\text {par}(C) :=\mathbb {R}(C-C)$ the smallest subspace parallel to *C*. Denote $\iota _{C}$ the indicator function of *C*, ${\mathcal {N}}_{C}$ the associated normal cone operator and $\text {P}_{C}$ the orthogonal projection on *C*. The strong relative interior of *C* is $\text {sri}(C)$.

*Functions* Given $R \in \Gamma _0(\mathcal {H})$, its subdifferential is a set-valued operator defined by $\partial R : \mathcal {H}\rightrightarrows \mathcal {H},\, x\mapsto \left\{ v \in \mathcal {H}: R(x') \ge R(x) + \langle v,\,x'-x \rangle , \forall x' \in \mathcal {H}\right\}$.

### Lemma 2.1

(Descent Lemma [26]) Suppose that $F: \mathcal {H}\rightarrow \mathbb {R}$ is convex continuously differentiable and $\nabla F$ is $(1/\beta )$-Lipschitz continuous. Then,

$$\begin{aligned} F(x) \le F(y) + \langle \nabla F(y),\,x-y \rangle + {\Vert } x-y {\Vert }^2,\quad \forall x,y\in \mathcal {H}. \end{aligned}$$

### Definition 2.1

(*Bregman Divergence*) Given a function $R \in \Gamma _0(\mathcal {H})$ and two points *x*, *y* in its effective domain $\text {dom}(R)$, the Bregman divergence is defined by

$$\begin{aligned} \mathcal {D}_{R}^{v} (y,x) :=R(y) - R(x) - \langle v,\,y-x \rangle , \end{aligned}$$

where $v \in \partial R(x)$ is a subgradient of *R*.

Notice that the Bregman divergence is not a distance in the usual sense, as it is in general not symmetric.^2^ However, it measures the distance of two points in the sense that $\mathcal {D}_{R}^{v} (x, x) = 0$ and $\mathcal {D}_{R}^{v} (y,x) \ge 0$ for any *x*, *y* in $\text {dom}(R)$. Moreover, $\mathcal {D}_{R}^{v} (y,x) \ge \mathcal {D}_{R}^{v} (w,x)$ for all *w* in the line segment between *x* and *y*.

*Operators* Given a set-valued mapping $A: \mathcal {H}\rightrightarrows \mathcal {H}$, define its graph as $\text {gph}\,(A) :=\{ (x,u)\in \mathcal {H}\times \mathcal {H}: \ u \in A(x) \}$, and set of zeros $\text {zer}(A) = \{ x \in \mathcal {H}: 0 \in A(x) \}$. Denote $(\text {Id}+ A)^{-1}$ the resolvent of *A*.

### Definition 2.2

(*Cocoercive Operator*) Let $\beta > 0$ and $B:\mathcal {H}\rightarrow \mathcal {H}$, then *B* is $\beta$-cocoercive, if $\langle B(x_1)-B(x_2),\,x_1-x_2 \rangle \ge \beta {\Vert } B(x_1)-B(x_2) {\Vert }^2 ,\, \forall x_1, x_2 \in \mathcal {H}$.

If an operator is $\beta$-cocoercive, then it is ${\beta }^{-1}$-Lipschitz continuous.

### Definition 2.3

(*Non-expansive Operator*) An operator $\mathscr {F}: \mathcal {H}\rightarrow \mathcal {H}$ is non-expansive, if ${\Vert } \mathscr {F}(x) - \mathscr {F}(y) {\Vert } \le {\Vert } x-y {\Vert },\,\forall x, y \in \mathcal {H}$. For any $\alpha \in ]0,1[$, $\mathscr {F}$ is called $\alpha$-averaged, if there exists a non-expansive operator $\mathscr {F}'$ such that $\mathscr {F}= \alpha \mathscr {F}' + (1-\alpha )\text {Id}$.

In particular, when $\alpha = \frac{1}{2}$, $\mathscr {F}$ is called *firmly non-expansive*. Several properties of firmly non-expansive operators are collected in the following lemma.

### Lemma 2.2

Let $\mathscr {F}: \mathcal {H}\rightarrow \mathcal {H}$, the following statements are equivalent:

(i) $\mathscr {F}$ is firmly non-expansive;
(ii) $2\mathscr {F}-\text {Id}$ is non-expansive;
(iii) $\mathscr {F}$ is the resolvent of a maximal monotone operator $A: \mathcal {H}\rightrightarrows \mathcal {H}$.

### Proof

$(\mathrm{i})\Leftrightarrow (\mathrm{ii})$ follows [1, Proposition 4.2, Corollary 4.29], and $(\mathrm{i})\Leftrightarrow (\mathrm{iii})$ is [1, Corollary 23.8]. $\square$

### Lemma 2.3

[1, Proposition 4.33] Let $F: \mathcal {H}\rightarrow \mathbb {R}$ be a convex differentiable function, with $\frac{1}{\beta }$-Lipschitz continuous gradient, $\beta \in ]0,+\infty [$, then $\text {Id}- \gamma \nabla F$ is $\frac{\gamma }{2\beta }$-averaged for $\gamma \in ]0,2\beta [$.

The next lemma shows the composition of two averaged operators.

### Lemma 2.4

[27, Theorem 3] Let $\mathscr {F}_1, \mathscr {F}_2: \mathcal {H}\rightarrow \mathcal {H}$ be $\alpha _1, \alpha _2$-averaged, respectively, then $\mathscr {F}_1 \circ \mathscr {F}_2$ is $\alpha$-averaged with $\alpha = \frac{\alpha _1+\alpha _2-2\alpha _1\alpha _2}{1-\alpha _1\alpha _2} \in ]0,1[$.

*Sequence* The following lemma is very classical, see *e.g.* [28, Theorem 3.3.1].

### Lemma 2.5

Let the non-negative sequence $\{a_k\}_{k\in \mathbb {N}}$ be non-increasing and summable. Then $a_{k}=o(k^{-1})$.

*Partial Smoothness* In this part, let $\mathcal {H}=\mathbb {R}^n$. We briefly introduce the concept of partial smoothness, which was introduced in [29] and lays the foundation of our local convergence analysis.

Let $\mathcal {M}$ be a $C^2$-smooth manifold of $\mathbb {R}^n$ around a point *x*. Denote $\mathcal {T}_{\mathcal {M}}(x')$ the tangent space to $\mathcal {M}$ at any point near *x* in $\mathcal {M}$; see Sect. 8 for more materials. Below we present the definition of partly smooth functions in $\Gamma _0(\mathbb {R}^n)$ setting.

### Definition 2.4

(*Partly Smooth Function*) Let $R \in \Gamma _0(\mathbb {R}^n)$, and $x\in \mathbb {R}^n$ such that $\partial R(x) \ne \emptyset$. *R* is then said to be *partly smooth* at *x* relative to a set $\mathcal {M}$ containing *x*, if

(i) *Smoothness*$\mathcal {M}$ is a $C^2$-manifold around *x*, $R|_{\mathcal {M}}$ is $C^2$ around *x*;
(ii) *Sharpness* The tangent space $\mathcal {T}_{\mathcal {M}}(x)$ coincides with $T_x :=\text {par}(\partial R(x))^\perp$;
(iii) *Continuity* The set-valued $\partial R$ is continuous at *x* relative to $\mathcal {M}$.

The class of partly smooth functions at *x* relative to $\mathcal {M}$ is denoted as $\text {PSF}_{x}(\mathcal {M})$.

Popular examples of partly smooth functions are summarized in Sect. 6 whose details can be found in [15].

## Problem and Algorithms

*Non-smooth Optimization* In this paper, we are interested in the following structured convex optimization problem

1 $$\begin{aligned} \min _{x\in \mathcal {H}} \left\{ F(x) + R(x):\, x \in V \right\} , \end{aligned}$$

where the following assumptions are imposed

1. *R* belongs to $$\Gamma _0(\mathcal {H})$$.
2. $F: \mathcal {H}\rightarrow \mathbb {R}$ is convex continuously differentiable with $\nabla F$ being $(1/\beta )$-Lipschitz continuous.
3. The constraint set *V* is a closed vector subspace of $\mathcal {H}$.
4. $\text {Argmin}_V(F+R)$ is non-empty and $0 \in \text {sri}(\text {dom}(R)-V)$.

Typical examples of (1) can be found in the numerical experiment section. These assumptions entail that $F+R+\iota _V \in \Gamma _0(\mathcal {H})$, and moreover, that

$$\begin{aligned} \text {zer}(\nabla F + \partial R + {\mathcal {N}}_{V}) = \text {zer}(\partial (F + R + \iota _V)) = \text {Argmin}_V(F+R) \ne \emptyset , \end{aligned}$$

using [1, Theorem 16.37(i)] and Fermat’s rule.

*Forward–Douglas–Rachford Splitting* When $V = \mathcal {H}$, problem (1) can be handled by the classical Forward–Backward splitting method [4], whose iteration, in its relaxed form, reads

2 $$\begin{aligned} x_{k+1}= (1-\lambda _{k}) x_{k}+ \lambda _{k}\text {prox}_{\gamma R} \left( {x_{k}- \gamma \nabla F(x_{k})}\right) , \end{aligned}$$

where $\gamma \in ]0, 2\beta [$ is the step size and $\lambda _{k}\in ]0, \frac{4\beta -\gamma }{2\beta }[$ is the relaxation parameter. The term $\text {prox}_{\gamma R}$ is called the proximity operator of $\gamma R$ and is defined by

3 $$\begin{aligned} \text {prox}_{\gamma R} (x) :=\text {argmin}_{u\in \mathcal {H}} \, \gamma R(u) + {\Vert } u-x {\Vert }^2 . \end{aligned}$$

When *V* is merely a subspace of $\mathcal {H}$, in principle we still can apply FB splitting method to solve (1). However, even if $\text {prox}_{\gamma R}$ is very easy to compute, the proximity operator of $R+\iota _{V}$ in general may be rather difficult to calculate. Therefore, new splitting algorithms are needed, and one possible choice is the Forward–Douglas–Rachford splitting method [2] which will be presented shortly. Let us first define $\text {P}_{V}$ as the orthogonal projector onto the subspace *V*, and the function $G :=F \circ \text {P}_{V}$. Then (1) is, obviously, equivalent to

4 $$\begin{aligned} \min _{x\in \mathcal {H}} \left\{ \Phi _{V}(x) :=G(x) + R(x) + \iota _{V}(x) \right\} . \end{aligned}$$

In turn, owing to Assumptions (A.1)–(A.4), we have

$$\begin{aligned} \emptyset \ne \text {Argmin}_V(F+R) = \text {Argmin}(\Phi _{V}) = \text {zer}(\nabla G + \partial R + {\mathcal {N}}_{V}) . \end{aligned}$$

### Remark 3.1

From the assumption on *F*, we have that also *G* is convex and continuously differentiable with $\nabla G = \text {P}_{V} \circ \nabla F \circ \text {P}_{V}$ being $(1/\beta _{{V}})$-Lipschitz continuous (notice that $\beta _{{V}}\ge \beta$). The observation of using *G* instead of *F* to achieve a better Lipschitz condition was first considered in [7].

The iteration of FDR method for solving (4) reads

5 $$\begin{aligned} \begin{aligned} u_{k+1}&= \text {prox}_{\gamma R} \left( { 2x_{k}- {z}_{k}- \gamma \nabla G (x_{k}) }\right) , \\ {z}_{k+1}&= {z}_{k}+ \lambda _{k}( u_{k+1}-x_{k}) , \\ x_{k+1}&= \text {P}_{V} ({z}_{k+1}) , \end{aligned} \end{aligned}$$

where $\gamma$ is step size and $\lambda _{k}$ is relaxation parameter. Recall that, under the conditions that $\gamma \in ]0, 2\beta _{{V}}[$, $\lambda _{k}\in ]0,\frac{4\beta _{{V}}- \gamma }{2\beta _{{V}}}[$ and $\sum _{k\in \mathbb {N}}\lambda _{k}\left( {\frac{4\beta _{{V}}-\gamma }{2\beta _{{V}}}-\lambda _{k}}\right) = +\infty$, the sequences $\{u_{k}\}_{k\in \mathbb {N}}, \,\{x_{k}\}_{k\in \mathbb {N}}$ converge to a solution; see [2, Theorem 4.2].

In this paper, we consider a non-stationary version of (5), namely $\gamma$ may change along the iterations. The method is described below in Algorithm 1.
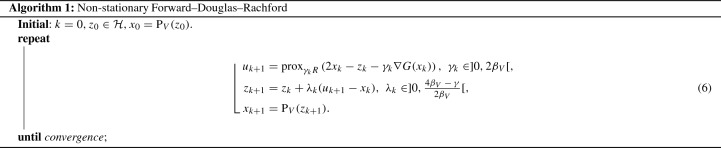


### Remark 3.2

For global convergence, one can also consider an inexact version of (6) by incorporating additive errors in the computation of $u_{k}$ and $x_{k}$, though we do not elaborate more on this for the sake of local convergence analysis. One can consult [8] for more details on this aspect.

In the next, we suppose the following main assumption on the parameters:

(A.5) The sequence of the step sizes $\{\gamma _{k}\}_{k\in \mathbb {N}}$ and the one of the relaxation parameters $\{\lambda _{k}\}_{k\in \mathbb {N}}$ verify:
  $0< \underline{\gamma }\le \gamma _{k}\le \overline{\gamma }< 2\beta _{{V}}$ and $\gamma _{k}\rightarrow \gamma$ for some $\gamma \in [\underline{\gamma },\overline{\gamma }]$;
  $$\lambda _{k}\in ]0, \frac{4\beta _{{V}}- \gamma _{k}}{2\beta _{{V}}}[$$ such that $$\sum _{k\in \mathbb {N}}\lambda _{k}(\frac{4\beta _{{V}}- \gamma _{k}}{2\beta _{{V}}}-\lambda _{k}) = +\infty $$;
  $$\sum _{k\in \mathbb {N}} \lambda _{k}{|} \gamma _{k}-\gamma {|} < +\infty $$.

Notice that, for the stationary case (*i.e.* for $\gamma _k$ constant), Assumption (A.5) is equivalent to the conditions required in [2, Theorem 4.2] for the convergence of iteration (5). Moreover, to satisfy (A.5) in the absence of relaxation (*i.e.* when the relaxation parameter is fixed to $\lambda _k\equiv 1$), the sequence of the step sizes has just to verify $\gamma _{k}\in ]\underline{\gamma },\overline{\gamma }[$ with $\sum _{k\in \mathbb {N}}{|} \gamma _{k}-\gamma {|} < +\infty$. On the other hand, in general, the summability assumption of $\{\lambda _{k}{|} \gamma _{k}-\gamma {|}\}_{k\in \mathbb {N}}$ in (A.5) is weaker than imposing it without $\lambda _k$. Indeed, following the discussion in [30, Remark 5.7], take $q\in ]0,1]$, let $\theta = \frac{4\beta _{{V}}- \overline{\gamma }}{4\beta _{{V}}} > \frac{1}{2}$ and

$$\begin{aligned} \lambda _k = {\theta - \sqrt{\theta -1/(2k)}}\quad \text { and }\quad {|} \gamma _k - \gamma {|} = (\theta + \sqrt{\theta -1/(2k)})/{k^q}. \end{aligned}$$

Then, it can be verified thatAs previously mentioned, FDR recovers DR [3] when $F = 0$, and FB [4] when $V = \mathcal {H}$. We briefly introduce below two other closely related operator splitting methods: the generalized Forward–Backward splitting (GFB) [6] and the three-operator splitting (TOS) [7].

*Generalized Forward–Backward Splitting* Let  be a positive integer. Now for problem (1), let $V = \mathcal {H}$ and suppose we have  non-smooth functionals. The problem then becomes: let  for each 7Similar to the situation of FDR algorithm, even if the proximity operator of each  can be solved easily, the proximity of the sum of them can be intractable. In [6], the authors propose the GFB algorithm, which achieves the full splitting of the evaluation of the proximity operator of each . Let  such that , choose $\gamma \in ]0, 2\beta [$ and $\lambda _{k}\in ]0,\frac{4\beta - \gamma }{2\beta }[$:8We refer to [6] for more details of the GFB algorithm. Now define the product space ${\varvec{\mathcal {H}}}:=\mathcal {H}\times \cdots \times \mathcal {H}$, equipped with proper inner product and norm, the subspace  and let the weights be . Then it can be shown that GFB algorithm is equivalent to applying FDR to the following problem:We refer to [2, 5] for more connections between FDR and GFB.

*Three-Operator Splitting* Let  in problem (7), then it becomes

9 $$\begin{aligned} \min _{x\in \mathcal {H}} F(x) + R_{1}(x) + R_{2}(x) . \end{aligned}$$

Notice that (9) can be handled by GFB as it is only a special case of (7). In [7] the author proposed a splitting scheme which resembles FDR yet different: given $\gamma \in ]0, 2\beta [$ and $\lambda _{k}\in ]0, \frac{4\beta - \gamma }{2\beta }[$, the iteration of TOS reads as follows:

10 $$\begin{aligned} \begin{aligned} u_{k+1}&= \text {prox}_{\gamma R_{1}} \left( { 2x_{k}- {z}_{k}- \gamma \nabla F (x_{k}) }\right) \\ {z}_{k+1}&= {z}_{k}+ \lambda _{k}( u_{k+1}-x_{k}) , \\ x_{k+1}&= \text {prox}_{\gamma R_{2}}({z}_{k+1}) . \end{aligned} \end{aligned}$$

It can be observed that the projection operator $\text {P}_{V}$ of FDR is replaced by the proximity operator $\text {prox}_{\gamma R_{2}}$. Though the difference is only for the update of $x_{k+1}$, their fixed-point operators are quite different; see in Sect. 5.4.

## Global Convergence

In this section, we deliver the global convergence analysis of the non-stationary FDR (6) in a general real Hilbert space setting, including convergence rate.

### Global Convergence of the Non-stationary FDR

Define the reflection operators of $\gamma R$ and $\iota _{V}$, respectively, as  and . Moreover, define the following operators:11Then the (stationary) FDR iteration (5) can be written into a fixed-point iteration in terms of ${z}_{k}$ [2, Theorem 4.2], namely

12 $$\begin{aligned} {z}_{k+1}= \mathscr {F}_{\gamma ,\lambda _k}({z}_{k}) . \end{aligned}$$

The next lemma shows the property of the fixed-point operator of FDR.

#### Lemma 4.1

For the FDR algorithm (6), let $\gamma \in ]0, 2\beta _{{V}}[$ and $\lambda _{k}\in ]0, \frac{4\beta _{{V}}-\gamma }{2\beta _{{V}}}[$. Then, we have that $\mathscr {F}_{\gamma }$ is $\frac{2\beta _{{V}}}{4\beta _{{V}}-\gamma }$-averaged and $\mathscr {F}_{\gamma ,\lambda _k}$ is $\frac{2\beta _{{V}}\lambda _{k}}{4\beta _{{V}}-\gamma }$-averaged.

#### Proof

The property of $\mathscr {F}_{\gamma }$ is a combination of Lemma 2.4 and [2, Proposition 4.1]. For $\mathscr {F}_{\gamma ,\lambda _k}$, it is sufficient to apply the definition of averaged operators. $\square$

Owing to [2, Theorem 4.2], under $\sum _{k\in \mathbb {N}}\lambda _{k}(\frac{4\beta _{{V}}-\gamma }{2\beta _{{V}}}-\lambda _{k}) = +\infty$ and conditions (A.1)–(A.4), $\{{z}_{k}\}_{k\in \mathbb {N}}$ converges weakly to some ${z}^\star \in \text {fix}(\mathscr {F}_{\gamma })$, and $\{x_{k}\}_{k\in \mathbb {N}}$ converges weakly to $x^{\star }:=\text {P}_{V}({z}^\star )$, where $\text {P}_{V}({z}^\star ) \in \text {Argmin}_V(F+R)$. On the other hand, the non-stationary FDR iteration (6) can be written as

13 $$\begin{aligned} \begin{aligned} {z}_{k+1}= \mathscr {F}_{\gamma _k,\lambda _k}({z}_{k})&= \left( {(1-\lambda _{k}){z}_{k}+ \lambda _{k}\mathscr {F}_{\gamma }({z}_{k})}\right) + \lambda _{k}\left( { \mathscr {F}_{\gamma _k}({z}_{k}) - \mathscr {F}_{\gamma }({z}_{k}) }\right) . \end{aligned} \end{aligned}$$

We are now ready to state our result on global convergence of Algorithm 1.

#### Theorem 4.1

Consider the non-stationary FDR iteration (6). Suppose that Assumptions (A.1)–(A.5) hold. Then, $\sum _{k\in \mathbb {N}}{\Vert } {z}_{k}-{z}_{k-1} {\Vert }^2 < +\infty$. Moreover, $\{{z}_{k}\}_{k\in \mathbb {N}}$ converges weakly to a point ${z}^\star \in \text {fix}(\mathscr {F}_{\gamma })$, and $\{x_{k}\}_{k\in \mathbb {N}}$ converges weakly to $x^{\star }:=\text {P}_{V}({z}^\star ) \in \text {Argmin}_V(F+R)$. If, in addition, either $\inf _{k \in \mathbb {N}} \lambda _k > 0$ or $\mathcal {H}$ is finite-dimensional, then $\{u_{k}\}_{k\in \mathbb {N}}$ converges weakly to $x^{\star }$.

The main idea of the proof of the theorem (see below) is to treat the non-stationarity as a perturbation error of the stationary iteration.

#### Remark 4.1

- As mentioned in the introduction, Theorem 4.1 remains true if the iteration is carried out inexactly, *i.e.* if $\mathscr {F}_{\gamma _k}({z}_{k})$ is computed approximately, provided that the errors are summable; see [8, Sect. 6] for more details.
- With more assumptions on how fast $\{\gamma _{k}\}_{k\in \mathbb {N}}$ converges to $\gamma$, we can also derive the convergence rate of the residuals $\{{\Vert } {z}_{k}-{z}_{k-1} {\Vert }\}_{k\in \mathbb {N}}$. However, as we will study in Sect. 5 local linear convergence behaviour of $\{{z}_{k}\}_{k\in \mathbb {N}}$, we shall forgo the discussion here. Interested readers can consult [8] for more details about the rate of residuals.

#### Proof

According to [8, Theorem 4], the following conditions are needed to ensure the convergence of the non-stationary iteration:

1. The set of fixed point of $\text {fix}(\mathscr {F}_{\gamma })$ is non-empty;
2. $\forall k\in \mathbb {N},\,\mathscr {F}_{\gamma _k}$ is 1-Lipschitz, *i.e.* non-expansive;
3. $$\lambda _k\in ]0, \frac{4\beta _V-\gamma _k}{2\beta _V}[$$ such that $$\inf _{k\in \mathbb {N}} \lambda _k (\frac{4\beta _V-\gamma _k}{2\beta _V}-\lambda _k) > 0$$;
4. $\forall \rho \in [0,+\infty [$ and $\Delta _{k,\rho } := \sup _{{\Vert } z {\Vert } \le \rho } {\Vert } \mathscr {R}_{\gamma _{k}}(z) - \mathscr {R}_{\gamma }(z) {\Vert }$ with $\mathscr {R}_{\gamma _{k}}, \mathscr {R}_{\gamma }$ being some non-expansive operators, there holds $\sum _{k} {\lambda _k\Delta _{k,\rho }} < +\infty$.

Owing to Lemma 4.1, given $\gamma _{k}\in [0, 2\beta _{{V}}]$, we have that $\mathscr {F}_{\gamma _k}$ is $\alpha _{k}$-averaged with $\alpha _{k} = \frac{2\beta _{{V}}}{4\beta _{{V}}-\gamma _{k}}$. This means that there exists a non-expansive operator $\mathscr {R}_{\gamma _{k}}$ such that $\mathscr {F}_{\gamma _k}= \alpha _{k}\mathscr {R}_{\gamma _{k}}+ (1-\alpha _{k})\text {Id}$. Similarly, for $\gamma \in [0, 2\beta _{{V}}]$, we have that $\mathscr {F}_{\gamma }$ is $\alpha$-averaged with $\alpha = \frac{2\beta _{{V}}}{4\beta _{{V}}-\gamma }$ and so that there exists a non-expansive operator $\mathscr {R}_{\gamma }$ such that $\mathscr {F}_{\gamma }= \alpha \mathscr {R}_{\gamma }+ (1-\alpha )\text {Id}$. Provided ${z}_{k}$, define the error term $e_{k}= (\mathscr {F}_{\gamma _k}-\mathscr {F}_{\gamma })({z}_{k})$. Then iteration (13) can be written as

14 $$\begin{aligned} \begin{aligned} {z}_{k+1}= (1-\lambda ){z}_{k}+ \lambda _{k}\mathscr {F}_{\gamma _k}({z}_{k})&= (1-\lambda ){z}_{k}+ \lambda _{k}\left( {\mathscr {F}_{\gamma }({z}_{k}) + e_{k}}\right) . \end{aligned} \end{aligned}$$

From Assumptions (A.1)–(A.5), we can derive the following results:

- We have $\text {Argmin}_V(F+R)=\text {zer}(\nabla G + \partial R + {\mathcal {N}}_{V})=\text {P}_{V}(\text {fix}(\mathscr {F}_{\gamma }))$ from the discussion of Assumptions (A.1)–(A.4). It then follows that $\text {fix}(\mathscr {F}_{\gamma }) \ne \emptyset$.
- Owing to Lemma 4.1, we have $\mathscr {F}_{\gamma _k,\lambda _k}$ is $(\alpha _{k}\lambda _{k})$-averaged non-expansive.
- Owing to the averageness of $\mathscr {F}_{\gamma }$ and $\mathscr {F}_{\gamma _k}$, we have
  $$\begin{aligned} \mathscr {R}_{\gamma }= \text {Id}+ \tfrac{1}{\alpha } (\mathscr {F}_{\gamma }- \text {Id}) \quad \text { and }\quad \mathscr {R}_{\gamma _{k}}= \text {Id}+ \tfrac{1}{\alpha _{k}} (\mathscr {F}_{\gamma _k}- \text {Id}) . \end{aligned}$$
  Let $\rho > 0$ be a positive number. Then, $\forall z \in \mathcal {H}$ such that ${\Vert } z {\Vert }\le \rho$,
  15 $$\begin{aligned} {\Vert } \mathscr {R}_{\gamma _{k}}(z) - \mathscr {R}_{\gamma }(z) {\Vert }= & {} {\Vert } \tfrac{1}{\alpha _{k}} (\mathscr {F}_{\gamma _k}- \text {Id}) (z) - \tfrac{1}{\alpha } (\mathscr {F}_{\gamma }- \text {Id}) (z) {\Vert } \nonumber \\\le & {} \tfrac{{|} \gamma _{k}-\gamma {|}}{2\beta _{{V}}}\left( {2\rho + {\Vert } \mathscr {F}_{\gamma }(0) {\Vert }}\right) + \tfrac{1}{\alpha _{k}} {\Vert } {\mathscr {F}_{\gamma _k}(z) - \mathscr {F}_{\gamma }(z)} {\Vert } .\nonumber \\ \end{aligned}$$
  Given $\gamma \in ]0, 2\beta _{{V}}[$, define the two operators  and $\mathscr {F}_{2,\gamma }= \text {Id}- \gamma \nabla G$. Then $\mathscr {F}_{1,\gamma }$ is firmly expansive (Lemma 2.2) and $\mathscr {F}_{2,\gamma }$ is $\frac{\gamma }{2\beta _{{V}}}$-averaged (Lemma 2.3). Now we have
  16 $$\begin{aligned} {\Vert } \mathscr {F}_{\gamma _k}(z) - \mathscr {F}_{\gamma }(z) {\Vert }\le & {} {\Vert } \mathscr {F}_{2,\gamma _{k}}(z) - \mathscr {F}_{2,\gamma }(z) {\Vert }\nonumber \\&+{\Vert } \mathscr {F}_{1,\gamma _{k}}\mathscr {F}_{2,\gamma }(z) - \mathscr {F}_{1,\gamma }\mathscr {F}_{2,\gamma }(z) {\Vert } . \end{aligned}$$
  For the first term of (16),
  17 $$\begin{aligned} \begin{aligned} {\Vert } \mathscr {F}_{2,\gamma _{k}}(z) - \mathscr {F}_{2,\gamma }(z) {\Vert }&= {|} \gamma _k-\gamma {|}{\Vert } \nabla G (z) {\Vert } \\ \text {(Triangle inequality and }\nabla G\text { is }\beta _{{V}}^{-1}\text {-Lip.)}&\le {|} \gamma _k-\gamma {|} (\beta _{{V}}^{-1}\rho + {\Vert } \nabla G(0) {\Vert }) , \end{aligned} \end{aligned}$$
  where $\nabla G(0)$ is obviously bounded. Now for the second term of (16), denote $z^{V}= \text {P}_{V}(z)$ and $z^{V^\bot }= z - z^{V}$, it can be derived that
  $$\begin{aligned} v = \mathscr {F}_{1,\gamma }\mathscr {F}_{2,\gamma }(z) \Longleftrightarrow v = z^{V^\bot }+ \text {prox}_{\gamma R}(z^{V}- z^{V^\bot }- \gamma \nabla G(z^{V})) . \end{aligned}$$
  Denote $y = z^{V}- z^{V^\bot }- \gamma \nabla G (z^{V})$. Then we have
  $$\begin{aligned} \mathscr {F}_{1,\gamma _{k}}\mathscr {F}_{2,\gamma }(z) - \mathscr {F}_{1,\gamma }\mathscr {F}_{2,\gamma }(z) = \text {prox}_{\gamma _k R}(y) - \text {prox}_{\gamma R}(y) . \end{aligned}$$
  Denote $w_k=\text {prox}_{\gamma _k R}(y)$ and $w=\text {prox}_{\gamma R}(y)$. Using the resolvent equation [31] and firm non-expansiveness of the proximity operator yields
  18 $$\begin{aligned} \begin{aligned} {\Vert } w_k-w {\Vert }&= {\Vert } \text {prox}_{\gamma _k R}(\tfrac{\gamma _k}{\gamma } y + \left( 1-\tfrac{\gamma _k}{\gamma }\right) w) - \text {prox}_{\gamma _k R}(y) {\Vert } \\&\le {\Vert } \left( 1-\tfrac{\gamma _k}{\gamma }\right) (y - w) {\Vert } = \tfrac{{|} \gamma _k-\gamma {|}}{\underline{\gamma }}{\Vert } y - w {\Vert } \\&\le \tfrac{{|} \gamma _k-\gamma {|}}{\underline{\gamma }}{\Vert } (\text {Id}- \text {prox}_{\gamma R})y {\Vert } \le \tfrac{{|} \gamma _k-\gamma {|}}{\underline{\gamma }} ({\Vert } y {\Vert }+{\Vert } \text {prox}_{\gamma R}(0) {\Vert }). \end{aligned} \end{aligned}$$
  Using the triangle inequality and non-expansiveness of $\beta _{{V}}\nabla G$, we obtain
  19 $$\begin{aligned} {\Vert } y {\Vert }&\le {\Vert } z^{V}- z^{V^\bot } {\Vert } + \gamma {\Vert } \nabla G(z^{V}) {\Vert } \le \rho + \gamma {\Vert } \nabla G(z^{V}) - \nabla G(0) {\Vert } + \gamma {\Vert } \nabla G(0) {\Vert } \nonumber \\&\le \rho + \gamma \beta _{{V}}^{-1} {\Vert } z {\Vert } + \gamma {\Vert } \nabla G(0) {\Vert } \le \rho + \overline{\gamma }\beta _{{V}}^{-1} \rho + \overline{\gamma }{\Vert } \nabla G(0) {\Vert } . \end{aligned}$$
  Define $\Delta _{k,\rho } :=\sup _{{\Vert } z {\Vert } \le \rho } {\Vert } \mathscr {R}_{\gamma _{k}}(z) - \mathscr {R}_{\gamma }(z) {\Vert }$. Then, putting together (15), (17), (18) and (19), we get that $\forall \rho \in [0,+\infty [$ where $C=\tfrac{2\rho +{\Vert } \mathscr {F}_{\gamma }(0) {\Vert }}{4\beta _{{V}}-\overline{\gamma }} + \tfrac{\rho }{\beta _{{V}}}(1 + \tfrac{\beta _{{V}}}{\underline{\gamma }} + \tfrac{\overline{\gamma }}{\underline{\gamma }} ) + (1 + \tfrac{\overline{\gamma }}{\underline{\gamma }}) {\Vert } \nabla G(0) {\Vert } + \tfrac{1}{\underline{\gamma }} {\Vert } \text {prox}_{\gamma R}(0) {\Vert }$ is finite valued.

To this point, we verified that all the conditions of [8, Theorem 4] are met for the non-stationary FDR. Weak convergence of the sequence $\{{z}_{k}\}_{k\in \mathbb {N}}$ then follows. In turn, since $\text {P}_{V}$ is linear, weak convergence of $\{x_{k}\}_{k\in \mathbb {N}}$ is also obtained.

For the sequence $\{u_{k}\}_{k\in \mathbb {N}}$, observe from the second equation in (6) that $u_{k+1}= ({z}_{k+1}-{z}_{k})/{\lambda _k} + x_{k}$, hence ${\Vert } u_{k+1}-x_{k} {\Vert } \le {{\Vert } {z}_{k+1}-{z}_{k} {\Vert }}/{\lambda _k}$. It follows from ${\Vert } {z}_{k+1}-{z}_{k} {\Vert } \rightarrow 0$ and the condition $\inf _{k \in \mathbb {N}} \lambda _k > 0$ that $u_{k+1}-x_{k}$ converges strongly to 0. We thus obtain weak convergence of $u_{k}$. If $\mathcal {H}$ is finite-dimensional, using (30) and the same argument as for inequality (18), we get ${\Vert } u_{k+1}-x^{\star } {\Vert } \le \tfrac{{|} \gamma _k-\gamma {|}}{\underline{\gamma }} ((2+\overline{\gamma }\beta _{{V}}){\Vert } x_{k}-x^{\star } {\Vert }+{\Vert } {z}_{k}-{z}^\star {\Vert }+{\Vert } \text {prox}_{\gamma R}(0) {\Vert }) \rightarrow 0$ which concludes the proof. $\square$

### Convergence Rate of the Bregman Divergence

In this part, we discuss the convergence rate of a specifically designed Bregman divergence associated to the objective value. As we have seen from the FDR iteration (5), there are three different points ${z}_{k}$ and $u_{k},x_{k}$ generated along the iteration, which makes very difficult to establish a convergence rate on the objective value directly, unless the constraint subspace *V* is the whole space. For instance, in [18] the author obtained an $o(1/\sqrt{k})$ convergence rate on $(R(u_{k})+G(x_{k}))-(R(x^{\star })+G(x^{\star }))$, which in general is *not* a non-negative quantity. Moreover, the functions *R* and *G* in the criterion are not evaluated at the same point. So the latter convergence rate is not only pessimistic (when specialized to $V=\mathcal {H}$ it gives a convergence rate as slow as subgradient descent), but is also of a limited interest given the lack of non-negativity. Our result in this part successfully avoids such drawbacks.

As in Theorem 4.1, let ${z}^\star \in \text {fix}(\mathscr {F}_{\gamma })$ and $x^{\star }:=\text {P}_{V}({z}^\star ) \in \text {Argmin}(\Phi _{V})$. Thus, (A.4) and Fermat’s rule allow to deduce that there exists a normal vector $v^{\star }\in V^\bot = {\mathcal {N}}_{V}(x^{\star })$ such that $v^{\star }\in \nabla G(x^{\star }) + \partial R(x^{\star })$. Now denote $\Phi :=R+G$. Recalling Definition 2.1, for $y \in \mathbb {R}^{n}$, define the following Bregman divergence to the solution $x^{\star }$

20 $$\begin{aligned} \mathcal {D}_{{\Phi }}^{v^{\star }}\!(y) :=\mathcal {D}_{\Phi }^{v^{\star }}(y,x^{\star })= & {} \Phi (y) - \Phi (x^{\star }) - \langle v^{\star },\,y-x^{\star } \rangle \nonumber \\= & {} \Phi (y)-\Phi (x^{\star }) - \langle v^{\star },\,y^{V^\bot } \rangle , \end{aligned}$$

where $y^{V^\bot }:=\text {P}_{V^\bot }(y)$ is the projection of *y* onto $V^\bot$. In the last equality, we used the trivial fact that $\langle v^{\star },\,x^{\star } \rangle =0$.

The motivation of choosing the above function to quantify the convergence rate of FDR algorithm is due to the fact that it measures both the discrepancy of the objective to the optimal value and violation of the constraint on *V*.

Lemma 4.2 hereafter will provide us a key estimate on $\mathcal {D}_{{\Phi }}^{v^{\star }}\!(u_{k})$ which will be used to derive the convergence rate of $\{\mathcal {D}_{{\Phi }}^{v^{\star }}\!(u_{k})\}_{k\in \mathbb {N}}$. Denote $z_{k}^{V^\bot }:=\text {P}_{V^\bot }({z}_{k})$ the projection of ${z}_{k}$ onto $V^\bot$, $\phi _{k}:=\frac{1}{2\gamma _{k}} ({\Vert } z_{k}^{V^\bot }+ \gamma _{k}v^{\star } {\Vert }^2 + {\Vert } x_{k}-x^{\star } {\Vert }^2)$ and two auxiliary quantities $\xi _{k}:=\tfrac{{|} \underline{\gamma }-\beta _{{V}} {|}}{2\underline{\gamma }\beta _{{V}}} {\Vert } {z}_{k}-{z}_{k-1} {\Vert }^2,\, \zeta _{k}:=\tfrac{{|} \gamma _{k}-\gamma _{k-1} {|}}{2\underline{\gamma }^2}{\Vert } {z}_{k}-x^{\star } {\Vert }^2$.

#### Lemma 4.2

Considering the non-stationary FDR iteration in (6). Suppose that Assumptions (A.1)–(A.5) hold with $\lambda _{k}\equiv 1$. Then,

(i) We have that $\mathcal {D}_{{\Phi }}^{v^{\star }}\!(y)\ge 0$ for every *y* in $\mathcal {H}$. Moreover, if *y* is a solution then $\mathcal {D}_{{\Phi }}^{v^{\star }}\!(y)=0$ (in particular, $\mathcal {D}_{{\Phi }}^{v^{\star }}\!(x^{\star })=0$). On the other hand, if *y* is feasible ($y\in V$) and $\mathcal {D}_{{\Phi }}^{v^{\star }}\!(y)=0$, then *y* is solution.
(ii) For the sequence $\{u_{k}\}_{k\in \mathbb {N}}$, if $v^{\star }$ is bounded we have
  21 $$\begin{aligned} \mathcal {D}_{{\Phi }}^{v^{\star }}\!(u_{k+1}) + \phi _{k+1}\le \phi _{k}+ {\Vert } v^{\star } {\Vert }^2 + \xi _{k+1}+ \zeta _{k+1}< +\infty . \end{aligned}$$

#### Remark 4.2

If we restrict $\gamma _{k}\in ]0, \beta _{{V}}]$, then the term $\xi _{k}$ in (21) can be discarded. If we assume $\{\gamma _{k}\}_{k\in \mathbb {N}}$ is monotonic, then the term $\zeta _{k}$ also disappears.

#### Proof

The non-negativity of $\mathcal {D}_{{\Phi }}^{v^{\star }}\!(u_{k})$ is rather obvious, as $\Phi$ is convex. Therefore, next we focus on the second claim. Define $y^{V^\bot }:=\text {P}_{V^\bot }(y)$, $u_{k}^{V^\bot }:=\text {P}_{V^\bot }(u_{k})$, $z_{k}^{V^\bot }:=\text {P}_{V^\bot }({z}_{k})$ the projections of $y, u_{k}, {z}_{k}$ onto $V^\bot$, respectively.

The update of $u_{k}$ in (6) and definition of proximity operator imply that

$$\begin{aligned} (2x_{k}- {z}_{k}- u_{k+1})/{\gamma _{k}} - \nabla G(x_{k}) \in \partial R(u_{k+1}) . \end{aligned}$$

For the convexity of *R*, we obtain that, for every $y \in \mathcal {H}$,

22 $$\begin{aligned} \begin{aligned} R(y)&\ge R(u_{k+1}) + \langle (2x_{k}- {z}_{k}- u_{k+1})/{\gamma _{k}} - \nabla G(x_{k}) ,\,y -u_{k+1} \rangle \\&= R(u_{k+1}) + \tfrac{1}{\gamma _{k}} \langle 2 x_{k}-{z}_{k}-u_{k+1},\,y -u_{k+1} \rangle - \langle \nabla G(x_{k}),\,y-u_{k+1} \rangle . \end{aligned} \end{aligned}$$

Notice that $u_{k+1}= x_{k}+ {z}_{k+1}-{z}_{k}$. Then, the first inner product of the last line of (22) can be rewritten as

23 $$\begin{aligned} \begin{aligned}&\langle 2x_{k}-{z}_{k}-u_{k+1},\,y-u_{k+1} \rangle \\&\quad = \langle x_{k}- {z}_{k},\,y-x_{k} \rangle + \langle y-{z}_{k},\,{z}_{k}-{z}_{k+1} \rangle + {\Vert } {z}_{k+1}-{z}_{k} {\Vert }^2 \\&\quad = -\langle z_{k}^{V^\bot },\,y \rangle + \tfrac{1}{2} \left( { {\Vert } {z}_{k+1}-{z}_{k} {\Vert }^2 + {\Vert } {z}_{k+1}-y {\Vert }^2 - {\Vert } {z}_{k}-y {\Vert }^2 }\right) , \end{aligned} \end{aligned}$$

where $2\langle c_2-c_1,\,c_1-c_3 \rangle = { {\Vert } c_2-c_3 {\Vert }^2 - {\Vert } c_1-c_2 {\Vert }^2 - {\Vert } c_1-c_3 {\Vert }^2 }$ is applied to $\langle y-{z}_{k},\,{z}_{k}-{z}_{k+1} \rangle$. Combining (23) with (22),

24 $$\begin{aligned} R(u_{k+1}) - R(y)\le & {} \langle \nabla G(x_{k}),\,y-u_{k+1} \rangle + \tfrac{1}{\gamma _{k}} \langle z_{k}^{V^\bot },\,y \rangle \nonumber \\&+ \tfrac{1}{2\gamma _{k}} \left( { {\Vert } {z}_{k}-y {\Vert }^2 - {\Vert } {z}_{k+1}-y {\Vert }^2 - {\Vert } {z}_{k+1}-{z}_{k} {\Vert }^2 }\right) . \end{aligned}$$

Since *G* is convex, given any $x_{k}$ and $y \in \mathcal {H}$, we have

25 $$\begin{aligned} G(x_{k}) - G(y) \le \langle \nabla G (x_{k}),\,x_{k}- y \rangle . \end{aligned}$$

Recall $\Phi = R + G$. Summing up (24) and (25) and rearranging the terms, then

$$\begin{aligned} \begin{aligned}&\left( {R(u_{k+1}) + G(x_{k}) }\right) - \Phi (y) + \tfrac{1}{2\gamma _{k}} \left( {{\Vert } {z}_{k+1}-y {\Vert }^2 - {\Vert } {z}_{k}-y {\Vert }^2 }\right) - \tfrac{1}{\gamma _{k}} \langle z_{k}^{V^\bot },\,y \rangle \\&\quad \le -\tfrac{1}{2\gamma _{k}} {\Vert } {z}_{k+1}-{z}_{k} {\Vert }^2 + \langle \nabla G(x_{k}),\,x_{k}-u_{k+1} \rangle . \end{aligned} \end{aligned}$$

Since *G* has Lipschitz continuous gradient, applying Lemma 2.1 yields

$$\begin{aligned} G(u_{k+1})- G(x_{k}) \le \langle \nabla G(x_{k}),\,u_{k+1}-x_{k} \rangle + \tfrac{1}{2\beta _{{V}}}{\Vert } u_{k+1}-x_{k} {\Vert }^2. \end{aligned}$$

Sum up the above two inequalities and recall $\xi _{k+1}:=\frac{{|} \underline{\gamma }-\beta _{{V}} {|}}{2\underline{\gamma }\beta _{{V}}} {\Vert } {z}_{k+1}-{z}_{k} {\Vert }^2$, then

26 $$\begin{aligned} \begin{aligned}&\Phi (u_{k+1}) - \Phi (y) + \tfrac{1}{2\gamma _{k}} \left( {{\Vert } {z}_{k+1}-y {\Vert }^2 - {\Vert } {z}_{k}-y {\Vert }^2 }\right) -\tfrac{1}{\gamma _{k}} \langle z_{k}^{V^\bot },\,y \rangle \\&\quad \le -\tfrac{1}{2\gamma _{k}} {\Vert } {z}_{k+1}-{z}_{k} {\Vert }^2 + \tfrac{1}{2\beta _{{V}}}{\Vert } u_{k+1}-x_{k} {\Vert }^2 \\&\quad = \tfrac{\gamma _{k}-\beta _{{V}}}{2\gamma _{k}\beta _{{V}}} {\Vert } {z}_{k+1}-{z}_{k} {\Vert }^2 \le \tfrac{{|} \gamma _{k}-\beta _{{V}} {|}}{2\gamma _{k}\beta _{{V}}} {\Vert } {z}_{k+1}-{z}_{k} {\Vert }^2 \le \xi _{k+1}. \end{aligned} \end{aligned}$$

Note that we applied again the equivalence $u_{k+1}= x_{k}+ {z}_{k+1}-{z}_{k}$. Furthermore, define $\zeta _{k+1}^{y} :=\frac{{|} \gamma _{k+1}-\gamma _{k} {|}}{2\underline{\gamma }^2}{\Vert } {z}_{k+1}-y {\Vert }^2$. Then, from (26), we have

27 $$\begin{aligned} \begin{aligned}&\Phi (u_{k+1}) + \tfrac{1}{2\gamma _{k+1}} {\Vert } {z}_{k+1}-y {\Vert }^2 \\&\quad = \Phi (u_{k+1}) + \tfrac{1}{2\gamma _{k}} {\Vert } {z}_{k+1}-y {\Vert }^2 + \left( {\tfrac{1}{2\gamma _{k+1}} - \tfrac{1}{2\gamma _{k}}}\right) {\Vert } {z}_{k+1}-y {\Vert }^2 \\&\quad \le \Phi (y) + \tfrac{1}{\gamma _{k}} \langle z_{k}^{V^\bot },\,y^{V^\bot } \rangle + \tfrac{1}{2\gamma _{k}} {\Vert } {z}_{k}-y {\Vert }^2 + \xi _{k+1}+ \zeta _{k+1}^{y} . \end{aligned} \end{aligned}$$

Recall that $x_{k}\in V$. Hence, $\text {P}_{V^\bot }(x_{k}) = 0$. Then, using (27), we have the following estimate for the Bregman divergence (defined in (20)):

$$\begin{aligned} \begin{aligned}&\mathcal {D}_{{\Phi }}^{v^{\star }}\!(u_{k+1}) -\mathcal {D}_{{\Phi }}^{v^{\star }}\!(y) = \Phi (u_{k+1}) - \Phi (x^{\star }) - \langle v^{\star },\,u_{k+1}^{V^\bot }-y^{V^\bot } \rangle \\&\quad \le \tfrac{1}{\gamma _{k}} \langle z_{k}^{V^\bot },\,y^{V^\bot } \rangle - \langle v^{\star },\,u_{k+1}^{V^\bot }-y^{V^\bot } \rangle + \tfrac{1}{2\gamma _{k}} {\Vert } {z}_{k}-y {\Vert }^2\\&\qquad -\tfrac{1}{2\gamma _{k+1}} {\Vert } {z}_{k+1}-y {\Vert }^2 + \xi _{k+1}+ \zeta _{k+1}^{y} \\&\quad = \tfrac{1}{2\gamma _{k}} \left( { {\Vert } y^{V^\bot }+\gamma _{k}v^{\star } {\Vert }^2 - 2{\Vert } \gamma _{k}v^{\star } {\Vert }^2 + {\Vert } z_{k}^{V^\bot }+\gamma _{k}v^{\star } {\Vert }^2 + {\Vert } z_{k}^{V}-y^{V} {\Vert }^2 }\right) \\&\qquad + \xi _{k+1}+ \zeta _{k+1}^{y} \\&\qquad + \tfrac{1}{2\gamma _{k+1}} \big ( - {\Vert } z_{k+1}^{V^\bot }+\gamma _{k+1}v^{\star } {\Vert }^2 + {\Vert } z_{k+1}^{V^\bot } {\Vert }^2 + {\Vert } \gamma _{k+1}v^{\star } {\Vert }^2 \\&\qquad - {\Vert } z_{k+1}^{V^\bot }-y^{V^\bot } {\Vert }^2 - {\Vert } z_{k+1}^{V}-y^{V} {\Vert }^2 \big ) , \end{aligned} \end{aligned}$$

where $y^{V}:=\text {P}_{V}(y), z_{k}^{V}:=\text {P}_{V}({z}_{k})$ are the projections of $y, {z}_{k}$ onto *V*, respectively.

From the above inequality, we deduce the following result

$$\begin{aligned} \begin{aligned}&\mathcal {D}_{{\Phi }}^{v^{\star }}\!(u_{k+1}) -\mathcal {D}_{{\Phi }}^{v^{\star }}\!(y) + \phi _{k+1}- \phi _{k}-(\xi _{k+1}+ \zeta _{k+1}^{y}) \\&\quad \le \tfrac{1}{2\gamma _{k}} \left( { {\Vert } y^{V^\bot }-\gamma _{k}v^{\star } {\Vert }^2 - 2{\Vert } \gamma _{k}v^{\star } {\Vert }^2 }\right) + \tfrac{1}{2\gamma _{k+1}} \left( { {\Vert } z_{k+1}^{V^\bot } {\Vert }^2 + {\Vert } \gamma _{k+1}v^{\star } {\Vert }^2 - {\Vert } z_{k+1}^{V^\bot }-y^{V^\bot } {\Vert }^2 }\right) \\&\quad = \tfrac{1}{2\gamma _{k}} \left( { {\Vert } y^{V^\bot } {\Vert }^2 - 2\gamma _{k}\langle y^{V^\bot },\,v^{\star } \rangle - {\Vert } \gamma _{k}v^{\star } {\Vert }^2 }\right) + \tfrac{1}{2\gamma _{k+1}} \left( { {\Vert } \gamma _{k+1}v^{\star } {\Vert }^2 - {\Vert } y^{V^\bot } {\Vert }^2 + 2\langle z_{k+1}^{V^\bot },\,y^{V^\bot } \rangle }\right) \\&\quad = \tfrac{\gamma _{k+1}-\gamma _{k}}{2\gamma _{k}\gamma _{k+1}} {\Vert } y^{V^\bot } {\Vert }^2 + \tfrac{\gamma _{k+1}- \gamma _{k}}{2} {\Vert } v^{\star } {\Vert }^2 + \tfrac{1}{\gamma _{k+1}}\langle z_{k+1}^{V^\bot }-\gamma _{k+1}v^{\star },\,y^{V^\bot } \rangle . \end{aligned} \end{aligned}$$

In particular, taking $y=x^{\star }\in V$ in the last inequality and using the fact that $\text {P}_{V^\bot }(x^{\star })=0$, we obtain the desired result. $\square$

With the above property of $\mathcal {D}_{{\Phi }}^{v^{\star }}\!(u_{k})$, we are able to present the main result on the convergence rate of the Bregman divergence.

#### Theorem 4.2

Consider the non-stationary FDR iteration (6). Suppose that Assumptions (A.1)–(A.5) hold with $\lambda _{k}\equiv 1$. If moreover $v^{\star }$ is bounded, then for any $k \ge 0$,

#### Remark 4.3

- A typical situation that ensures the boundedness of $v^{\star }$ is when $\partial R(x^{\star })$ is bounded. Such requirement can be removed if we choose more carefully the element $v^{\star }$. For instance, one can easily show from Theorem 4.1 that the subgradient $v_k :=(x_{k}-{z}_{k})/\gamma _k = -\text {P}_{V^\bot }(z_k)/\gamma _k$ converges weakly to $v^{\star }:=(x^{\star }- {z}^\star )/\gamma \in V^\bot \cap (\nabla G(x^{\star }) + \partial R(x^{\star }))$.
- The main difficulty in establishing the convergence rate directly on $\mathcal {D}_{{\Phi }}^{v^{\star }}\!(u_{k})$ (rather that on the best iterate) is that, for $V \subsetneq \mathcal {H}$, we have no theoretical guarantee that ${\mathcal {D}_{{\Phi }}^{v^{\star }}\!(u_{k})}$ is decreasing, *i.e.*  no descent property on $\mathcal {D}_{{\Phi }}^{v^{\star }}\!(u_{k})$.

#### Proof

Define $\theta _k :=\min _{0 \le i \le k} \mathcal {D}_{{\Phi }}^{v^{\star }}\!(u_{i}) \le \mathcal {D}_{{\Phi }}^{v^{\star }}\!(u_{k})$. Summing inequality (21) up to some $k \in \mathbb {N}$ yieldsSince $v^{\star }$ is bounded, so is $\phi _{0}$. Then, owing to Theorem 4.1, we haveLastly, as $\{{z}_{k}\}_{k\in \mathbb {N}}$ is bounded, so is $\{{\Vert } {z}_{k}-x^{\star } {\Vert }\}_{k\in \mathbb {N}}$. Recall that, by assumptions, $\{\gamma _{k}\}_{k\in \mathbb {N}}$ converges to some $\gamma \in ]0, 2\beta _{{V}}[$ with $\{{|} \gamma _{k}-\gamma {|}\}_{k\in \mathbb {N}}$ being summable. ThenSumming up the above results, we have that $(k+1)\theta _k \le C < +\infty$ holds for all $k \in \mathbb {N}$, which means $\theta _k = O(1/(k+1))$. Now, owing to the definition of $\theta _{k}$,Moreover, it is immediate that, for every $k \ge 1$,

$$\begin{aligned} \theta _{k}=\min (\mathcal {D}_{{\Phi }}^{v^{\star }}\!(u_{k}),\theta _{k-1}) \le \theta _{k-1} , \end{aligned}$$

that is, the sequence $\{\theta _k\}_{k\in \mathbb {N}}$ is non-increasing. Invoking Lemma 2.5 on $\{\theta _k\}_{k\in \mathbb {N}}$ concludes the proof.

For the ergodic rate, we start again from (21) and apply Jensen’s inequality to $\mathcal {D}_{{\Phi }}^{v^{\star }}\!$ which is a convex function, and getwhere the right-hand side is bounded by arguing as above. $\square$

### Application to Forward–Backward Splitting

Assume now that $V = \mathcal {H}$, in which case problem (4) simplifies to

$$\begin{aligned} \min _{x\in \mathcal {H}} \left\{ \Phi (x) {:=} F(x) + R(x) \right\} . \end{aligned}$$

In this case, the FDR iteration (6) is nothing but the FB splitting scheme (2). The non-relaxed and non-stationary version of it reads as

28 $$\begin{aligned} x_{k+1}= \text {prox}_{\gamma _{k} R} \left( { x_{k}- \gamma _{k} \nabla F (x_{k}) }\right) . \end{aligned}$$

We get $\mathcal {D}_{{\Phi }}^{v^{\star }}\!(y) = \Phi (y) - \Phi (x^{\star })$ by specializing the Bregman divergence (20) to $\Phi$, which is simply the objective value error. We have the following result.

#### Corollary 4.1

Consider the Forward–Backward iteration (28). Suppose that conditions (A.1)–(A.5) hold with $V = \mathcal {H}$ and $\lambda _k \equiv 1$. Then

$$\begin{aligned} \Phi (x_{k})-\Phi (x^{\star }) = o(1/k). \end{aligned}$$

#### Remark 4.4

- The *o*(1 / *k*) convergence rate for the large choice $\gamma _{k} \in ]0, 2\beta [$ appears to be new for the FB splitting algorithm. The rate *O*(1 / *k*) is known in the literature for several choices of the step size; see, for example, [12, Theorem 3.1] for $\gamma _k \in ]0,\beta ]$ or with backtracking, and [11, Proposition 2] for $\gamma _{k} \in ]0, 2\beta [$.
- For the global convergence of the sequence $\{x_{k}\}_{k\in \mathbb {N}}$ generated by the non-stationary FB iteration, neither convergence of $\gamma _{k}$ to $\gamma$ nor summability of $\{{|} \gamma _{k}-\gamma {|}\}_{k\in \mathbb {N}}$ is required. See [32, Theorem 3.4].

#### Proof

First, weak convergence of the non-stationary FB iteration follows from Theorem 4.1. On the one hand, specializing (21) to the case of FB, we get

29 $$\begin{aligned} \Phi (x_{k+1}) - \Phi (x^{\star })\le & {} \tfrac{1}{2\gamma _{k}} {\Vert } x_{k}-x^{\star } {\Vert }^2 - \tfrac{1}{2\gamma _{k+1}} {\Vert } x_{k+1}-x^{\star } {\Vert }^2 \nonumber \\&+ \tfrac{{|} \underline{\gamma }-\beta {|}}{2\underline{\gamma }\beta } {\Vert } x_{k}-x_{k-1} {\Vert }^2 + \tfrac{{|} \gamma _{k+1}-\gamma _{k} {|}}{2\underline{\gamma }^2}{\Vert } x_{k+1}-x^{\star } {\Vert }^2 , \end{aligned}$$

which means thatOn the other hand, owing to inequality (26) in the proof of Lemma 4.2, $\forall y \in \mathcal {H}$,

$$\begin{aligned} \Phi (x_{k+1}) + \tfrac{1}{2\gamma _k} {\Vert } x_{k+1}-y {\Vert }^2 \le \Phi (y) + \tfrac{1}{2\gamma _k} {\Vert } x_{k}-y {\Vert }^2+\left( {\tfrac{1}{2\beta }-\tfrac{1}{2\gamma _k}}\right) {\Vert } x_{k+1}-x_{k} {\Vert }^2 . \end{aligned}$$

Choosing $y=x_{k}$, we obtain

$$\begin{aligned} \begin{aligned} \left( {\Phi (x_{k+1}) - \Phi (x^{\star })}\right) - \left( {\Phi (x_{k}) - \Phi (x^{\star }) }\right)&\le \left( {\tfrac{1}{2\beta }-\tfrac{1}{\gamma _k}}\right) {\Vert } x_{k+1}-x_{k} {\Vert }^2 \\&\le -\delta {\Vert } x_{k+1}-x_{k} {\Vert }^2 , \end{aligned} \end{aligned}$$

where $\delta =\frac{1}{\overline{\gamma }}-\frac{1}{2\beta } > 0$ since $\overline{\gamma }< 2\beta$. This implies that the sequence $\{\Phi (x_{k}) - \Phi (x^{\star })\}_{k\in \mathbb {N}}$ is positive and non-increasing. Summing up both sides of the above inequality and applying Lemma 2.5 leads to the claimed result. $\square$

## Local Linear Convergence

From now on, we turn to the local convergence analysis of FDR. Given that partial smoothness is so far available only in finite dimension, in this section, we consider a finite-dimensional setting, *i.e.*$\mathcal {H}=\mathbb {R}^{n}$. In the sequel, we denote ${z}^\star \in \text {fix}(\mathscr {F}_{\gamma })$ a fixed point of iteration (6) and $x^{\star }= \text {P}_{V}({z}^\star ) \in \text {Argmin}(\Phi _{V})$ a global minimizer of problem (4). For simplicity, we also fix $\lambda _k \equiv 1$.

### Finite Activity Identification

We start with the finite activity identification, which means that in a finite number of iterations the iterates identify the manifold in which the solution $x^{\star }$ lives. Under the condition of Theorem 4.1, we know that $\gamma _{k}\rightarrow \gamma$, ${z}_{k}\rightarrow {z}^\star$ and $u_{k},x_{k}\rightarrow x^{\star }$. Moreover, we have the following optimality conditions

30 $$\begin{aligned} (x^{\star }- {z}^\star )/{\gamma } \in \nabla G (x^{\star }) + \partial R(x^{\star }) \quad \text { and }\quad ({z}^\star - x^{\star })/{\gamma } \in V^\bot , \quad x^{\star }\in V . \end{aligned}$$

The condition needed for identification result is built upon these monotone inclusions. Since $x_{k}$ is the projection of ${z}_{k}$ onto *V*, we have $x_{k}\in V$ for all $k \ge 0$. Therefore, we only need to discuss the identification property of $u_{k}$.

#### Theorem 5.1

For the non-stationary FDR (6). Suppose that Assumptions (A.1)–(A.5) hold, so that $(u_{k},x_{k},{z}_{k}) \rightarrow (x^{\star },x^{\star },{z}^\star )$ where ${z}^\star \in \text {fix}(\mathscr {F}_{\gamma })$ and $x^{\star }= \text {P}_{V}({z}^\star ) \in \text {Argmin}(\Phi _{V})$. Moreover, suppose that $R \in \text {PSF}_{x^{\star }}(\mathcal {M}_{x^{\star }}^{R})$ and that the following non-degeneracy condition holds

31 $$\begin{aligned} (x^{\star }- {z}^\star )/{\gamma } - \nabla G (x^{\star }) \in \text {ri}\left( { \partial R(x^{\star }) }\right) . \end{aligned}$$

Then,

(i) There exists $K \in \mathbb {N}$ such that, for all $k \ge K$, we have $u_{k}\in \mathcal {M}_{x^{\star }}^{R}$.
(ii) Moreover, for every $k \ge K$,
  - If $$\mathcal {M}_{x^{\star }}^{R}= x^{\star }+T_{x^{\star }}^R$$, then $$T_{u_{k}}^R=T_{x^{\star }}^R$$.
  - if *R* is locally polyhedral around $x^{\star }$, then $x_{k}\in \mathcal {M}_{x^{\star }}^{R}=x^{\star }+T_{x^{\star }}^R$, $T_{u_{k}}^R=T_{x^{\star }}^R$, $\nabla _{\mathcal {M}_{x^{\star }}^{R}} R(u_{k})=\nabla _{\mathcal {M}_{x^{\star }}^{R}} R(x^{\star })$, and $\nabla ^2_{\mathcal {M}_{x^{\star }}^{R}} R(u_{k})=0$.

#### Remark 5.1

As we mentioned before, for global convergence, approximation errors can be allowed, *i.e.*$\text {prox}_{\gamma R}$ and $\nabla G$ can be computed approximately. However, for the finite activity, we have no identification guarantees for $(u_{k}, x_{k})$ if such an approximation is allowed. For example, if we have $x_{k}= \text {P}_{V}({z}_{k}) + \varepsilon _{k}$ where $\varepsilon _{k}\in \mathbb {R}^{n}$ is the error of approximating $\text {P}_{V}({z}_{k})$. Then, unless $\varepsilon _{k} \in V$, we can no longer guarantee that $x_{k}\in V$.

#### Proof

From the update of $u_{k+1}$ and the definition of proximity operator, we have $(2x_{k}-{z}_{k}- u_{k+1})/{\gamma _{k}} - \nabla G(x_{k}) \in \partial R ( u_{k+1})$. At convergence, we have $(x^{\star }- {z}^\star )/{\gamma } - \nabla G(x^{\star }) \in \partial R ( x^{\star })$. Therefore, one can show that

$$\begin{aligned} \begin{aligned}&\text {dist}\left( {(x^{\star }-{z}^\star )/\gamma - \nabla G(x^{\star }), \partial R (u_{k+1})}\right) \\&\quad \le \tfrac{1}{\underline{\gamma }}(2{\Vert } x_{k}-x^{\star } {\Vert } \!+\! {\Vert } u_{k+1}-x^{\star } {\Vert } \!+\! {\Vert } {z}_{k}-{z}^\star {\Vert }) + \tfrac{{|} \gamma _{k}-\gamma {|}}{\underline{\gamma }^2} {\Vert } \text {P}_{V^\bot }({z}^\star ) {\Vert } + \tfrac{1}{\beta _{{V}}}{\Vert } x_{k}-x^{\star } {\Vert } . \end{aligned} \end{aligned}$$

Theorem 4.1 allows to infer that the right-hand side of the inequality converges to 0. In addition, since $R \in \Gamma _0(\mathbb {R}^{n})$, *R* is subdifferentially continuous at every point in its domain [33, Example 13.30], and in particular at $x^{\star }$. It then follows that $R(u_{k}) \rightarrow R(x^{\star })$. Altogether, this shows that the conditions of [34, Theorem 5.3] are fulfilled for *R*: (1) convergence of sequence; (2) distance $\text {dist}((x^{\star }-{z}^\star )/\gamma - \nabla G(x^{\star }), \partial R (u_{k+1})) \rightarrow 0$; (3) convergence of objective function value. The finite identification claim follows.

- In this case, $\mathcal {M}_{x^{\star }}^{R}$ is an affine subspace, *i.e.*$\mathcal {M}_{x^{\star }}^{R}= x^{\star }+T_{x^{\star }}^R$. Since *R* is partly smooth at $x^{\star }$ relative to $\mathcal {M}_{x^{\star }}^{R}$, the sharpness property holds at all nearby points in $\mathcal {M}_{x^{\star }}^{R}$ [29, Proposition 2.10]. Thus, for *k* large enough, *i.e.*$u_{k}$ sufficiently close to $x^{\star }$ on $\mathcal {M}_{x^{\star }}^{R}$, we have $\mathcal {T}_{u_{k}}(\mathcal {M}_{x^{\star }}^{R})=T_{x^{\star }}^R=T_{u_{k}}^R$.
- It is immediate to verify that a locally polyhedral function around $x^{\star }$ is indeed partly smooth relative to the affine subspace $x^{\star }+T_{x^{\star }}^R$. Thus, the first claim follows from (ii)(a). For the rest, it is sufficient to observe that by polyhedrality, for any $x \in \mathcal {M}_{x^{\star }}^{R}$ near $x^{\star }$, $\partial R(x) = \partial R(x^{\star })$. Therefore, combining local normal sharpness [29, Proposition 2.10] and [15, Lemma 4.3] yields the second conclusion.

$$\square $$

*A Bound on the Number of Iterations to Identification* In Theorem 5.1, we only assert the existence of some $K \ge 0$ beyond which finite identification occurs. There are situations where a bound of *K* can be established.

#### Proposition 5.1

Suppose that the assumptions of Theorem 5.1 hold. If the iterates are such that $\partial R(u_{k}) \subset \text {rbd}(\partial R(x^{\star }))$ whenever $u_{k}\notin \mathcal {M}_{x^\star }$, then we have $u_{k}\in \mathcal {M}_{x^\star }$ for some *k* obeying $k \ge \frac{{\Vert } z_0 - {z}^\star {\Vert }^2 + O(\sum _{k\in \mathbb {N}}{|} \gamma _k-\gamma {|})}{\underline{\gamma }^{2} \text {dist}(- \nabla G (x^{\star }), V^\bot + \text {rbd}(\partial R(x^{\star })))^2}$.

#### Remark 5.2

When $V = \mathbb {R}^n$, we recover the result of [15, Proposition 3.6(i)] established for the Forward–Backward splitting method. For $F=0$, our result also encompasses that of Douglas–Rachford splitting [16, Proposition 5.1].

#### Proof

Recall from the proof of Theorem 4.1 that $\mathscr {F}_{\gamma }{:=} \mathscr {F}_{1,\gamma }\circ \mathscr {F}_{2,\gamma }$, where  and $\mathscr {F}_{2,\gamma }= (\text {Id}- \gamma \nabla G )$. From (14), we have ${z}_{k+1}= \mathscr {F}_{\gamma }({z}_{k}) + e_{k}$ where $\{{\Vert } e_{k} {\Vert }\}_{k\in \mathbb {N}} = \{{|} \gamma _k - \gamma {|}\}_{k\in \mathbb {N}}$ is a summable sequence. Thus, arguing as in [35, Theorem 3.1], and using firm non-expansiveness of $\mathscr {F}_{1,\gamma }$ (Lemma 2.2) and non-expansiveness of $\mathscr {F}_{2,\gamma }$ (Lemma 2.3), we get

32 $$\begin{aligned} {\Vert } {z}_{k}-{z}^\star {\Vert }^2= & {} {\Vert } \mathscr {F}_{\gamma _k}({z}_{k-1}) - \mathscr {F}_{\gamma _k}({z}^\star ) {\Vert }^2 \le {\Vert } \mathscr {F}_{\gamma }({z}_{k-1}) - \mathscr {F}_{\gamma }({z}^\star ) {\Vert }^2 + O({\Vert } e_{k-1} {\Vert }) \nonumber \\= & {} {\Vert } {z}_{k-1}- {z}^\star {\Vert }^2 - {\Vert } g_{k}+ v_{k-1}+ \gamma \nabla G (x^{\star }) {\Vert }^2 + O({\Vert } e_{k-1} {\Vert }) , \end{aligned}$$

where $g_{k}{:=} 2x_{k-1}- {z}_{k-1}- u_{k}- \gamma \nabla G (x_{k-1})$ which verifies $g_k \in \gamma \partial R(u_{k})$ and $v_{k-1}:={z}_{k-1}-x_{k-1}\in V^\bot$. Assume that identification has not occurred yet, *i.e.*$u_{k}\notin \mathcal {M}_{x^\star }$ which implies $g_{k}+ v_{k-1}\in V^\bot + \partial R(u_{k}) \subset V^\bot + \text {rbd}(\partial R(x^{\star }))$.

Thus, continuing (32), we getNote $\text {dist}(-\nabla G(x^{\star }), V^\bot + \text {rbd}(\partial R(x^{\star }))) > 0$ since $-\nabla G (x^{\star }) \in \text {ri}(V^\bot + \partial R(x^{\star }))$ by (31). Taking *k* as the largest integer such that the right-hand side is positive, we deduce that the number of iterations where identification has not occurred does not exceed the claimed bound. Thus, finite identification necessarily occurs at some *k* larger than this bound. $\square$

### Locally Linearized Iteration

With the finite identification result, in the next we show that the globally nonlinear fixed-point iteration (13) can be locally linearized along the identified manifold $\mathcal {M}_{x^{\star }}^{R}$. Define the function $\overline{R}(u) {:=} \gamma R(u) - \langle u,\,x^{\star }-{z}^\star -\gamma \nabla G(x^{\star }) \rangle$. We have the following key property of $\overline{R}$.

#### Lemma 5.1

Let $x^{\star }\in \text {Argmin}(\Phi _{V})$, and suppose that $R \in \text {PSF}_{x^{\star }}(\mathcal {M}_{x^{\star }}^{R})$. Then the Riemannian Hessian of $\overline{R}$ at $x^{\star }$ reads as

33 $$\begin{aligned} {H}_{\overline{R}}{:=} \text {P}_{T^R_{x^{\star }}}\nabla ^2_{\mathcal {M}_{x^{\star }}^{R}} \overline{R}(x^{\star }) \text {P}_{T^R_{x^{\star }}}, \end{aligned}$$

which is symmetric positive semi-definite under either of the two conditions:

(i) Condition (31) holds.
(ii) $\mathcal {M}_{x^{\star }}^{R}$ is an affine subspace.

In turn, the matrix ${W}_{\overline{R}}{:=} (\text {Id}+{H}_{\overline{R}})^{-1}$ is firmly non-expansive.

#### Proof

See [15, Lemma 4.3] and [1, Corollary 4.3(ii)]. $\square$

From now on, we assume that *F* (hence *G*) is locally $C^2$-smooth around $x^{\star }$. Define ${H}_{G}{:=} \text {P}_{V}\nabla ^2 F(x^{\star }) \text {P}_{V}$, ${M}_{\overline{R}}{:=} \text {P}_{T^R_{x^{\star }}}{W}_{\overline{R}}\text {P}_{T^R_{x^{\star }}}$ and  andand $\mathscr {M}_{\gamma ,\lambda }= (1-\lambda ) \text {Id}+ \lambda \mathscr {M}_{\gamma }$. We have the following theorem for the linearized fixed-point formulation of (6).

#### Theorem 5.2

Consider the non-stationary FDR iteration (6) and suppose that (A.1)–(A.5) hold. If moreover, $\lambda _{k}\rightarrow \lambda \in ]0, \frac{4\beta _{{V}}-\gamma }{2\beta _{{V}}}[$ and *F* is locally $C^2$ around $x^{\star }$, then for all *k* large enough we have

34 $$\begin{aligned} {z}_{k+1}- {z}^\star = \mathscr {M}_{\gamma ,\lambda }({z}_{k}- {z}^\star ) + \psi _{k}+ \chi _{k}, \end{aligned}$$

where $\psi _{k}{:=} o({\Vert } {z}_{k}-{z}^\star {\Vert })$ and $\chi _{k}{:=} O(\lambda _{k}{|} \gamma _{k}-\gamma {|})$. Both $\psi _{k}$ and $\chi _{k}$ vanish when *R* is locally polyhedral around $x^{\star }$, *F* is quadratic and $(\gamma _{k}, \lambda _{k}) \in ]0, 2\beta _{{V}}[ \times ]0, \frac{4\beta _{{V}}-\gamma }{2\beta _{{V}}}[$ are chosen constants.

#### Proof

From (6), since *V* is a subspace, then we have

$$\begin{aligned} x_{k}= \text {P}_{V} ({z}_{k}) ,\, x^{\star }= \text {P}_{V} ({z}^\star ) \,\iff \, {z}_{k}- x_{k}\in {\mathcal {N}}_{V}(x_{k}) , \, {z}^\star - x^{\star }\in {\mathcal {N}}_{V}(x^{\star }) . \end{aligned}$$

Projecting onto *V* leads to $x_{k}- x^{\star }= \text {P}_{V} ({z}_{k}- {z}^\star )$. Under the assumptions of Theorem 5.1, there exists $K \in \mathbb {N}$ large enough such that for all $k \ge K$, $u_{k}\in \mathcal {M}_{x^{\star }}^{R}$. Denote $T_{u_{k}}^R$ and $T_{x^{\star }}^R$ the tangent spaces corresponding to $u_{k}$ and $x^{\star }\in \mathcal {M}_{x^{\star }}^{R}$. Denote $\tau _{k}^{R} : T_{u_{k}}^R \rightarrow T_{x^{\star }}^R$ the parallel translation along the unique geodesic on $\mathcal {M}_{x^{\star }}^{R}$ joining $u_{k}$ to $x^{\star }$. Owing to [19, Lemma 5.1], we have for $u_{k}$ after identification that $u_{k}-x^{\star }= \text {P}_{T^R_{x^{\star }}}(u_{k}-x^{\star }) + o({\Vert } u_{k}-x^{\star } {\Vert })$. The update of $u_{k+1}$ in (6) and its convergence are, respectively, equivalent to

$$\begin{aligned} \begin{aligned} {2x_{k}-{z}_{k}- u_{k+1}} - \gamma _{k}\nabla G(x_{k})&\in \gamma _{k}\partial R ( u_{k+1}) \\ {2x^{\star }-{z}^\star - x^{\star }} - \gamma \nabla G(x^{\star })&\in \gamma \partial R(x^{\star }) . \end{aligned} \end{aligned}$$

Upon projecting onto the corresponding tangent spaces and applying the parallel translation $\tau _{k+1}$ from $u_{k+1}$ to $x^{\star }$, we get

$$\begin{aligned} \gamma _{k}\tau _{k+1} \nabla _{\mathcal {M}_{x^{\star }}^{R}} R(u_{k+1})= & {} \text {P}_{T^R_{x^{\star }}} \left( {2x_{k}- {z}_{k}- u_{k+1}- \gamma _{k}\nabla G(x_{k}) }\right) \\&+ \left( {\tau _{k+1} \text {P}_{T^R_{u_{k+1}}} - \text {P}_{T^R_{x^{\star }}}}\right) \left( {2x_{k}- {z}_{k}- u_{k+1}- \gamma _{k}\nabla G(x_{k}) }\right) , \\ \gamma \nabla _{\mathcal {M}_{x^{\star }}^{R}} R(x^{\star })= & {} \text {P}_{T^R_{x^{\star }}} \left( {2x^{\star }- {z}^\star - x^{\star }- \gamma \nabla G(x^{\star })}\right) . \end{aligned}$$

Subtracting both equations, we obtain

35 $$\begin{aligned}&\gamma _{k}\tau _{k+1} \nabla _{\mathcal {M}_{x^{\star }}^{R}} R(u_{k+1}) - \gamma \nabla _{\mathcal {M}_{x^{\star }}^{R}} R(x^{\star }) \nonumber \\&\quad = \text {P}_{T^R_{x^{\star }}}\left( {(2x_{k}- {z}_{k}- u_{k+1}- \gamma _{k}\nabla G(x_{k}) ) - (2x^{\star }- {z}^\star - x^{\star }- \gamma \nabla G(x^{\star }) )}\right) \nonumber \\&\qquad + \mathbf{Term 1 } + \mathbf{Term 2 }, \end{aligned}$$

where we have $\mathbf{Term 1 } = (\tau _{k+1} \text {P}_{T^R_{u_{k+1}}} - \text {P}_{T^R_{x^{\star }}}) (x^{\star }- {z}^\star - \gamma \nabla G(x^{\star }) )$ and $\mathbf{Term 2 } = (\tau _{k+1} \text {P}_{T^R_{u_{k+1}}} - \text {P}_{T^R_{x^{\star }}}) ((2x_{k}- {z}_{k}- u_{k+1}- \gamma _{k}\nabla G(x_{k}) ) - (2x^{\star }- {z}^\star - x^{\star }- \gamma \nabla G(x^{\star }) ))$. For the term $(\gamma _{k}-\gamma ) \tau _{k+1} \nabla _{\mathcal {M}_{x^{\star }}^{R}} R(u_{k+1})$, since the Riemannian gradient $\nabla _{\mathcal {M}_{x^{\star }}^{R}} R(u_{k+1})$ is bounded on a bounded set, we have $(\gamma _{k}-\gamma ) \tau _{k+1} \nabla _{\mathcal {M}_{x^{\star }}^{R}} R(u_{k+1}) = O({|} \gamma _{k}-\gamma {|})$. For **Term 2**, owing to [15, Lemma B.1] and the boundedness of $\nabla G$, we have

$$\begin{aligned} \begin{aligned} \mathbf{Term 2 }&= o({\Vert } (2x_{k}- {z}_{k}- u_{k+1}- \gamma _{k}\nabla G(x_{k}) ) - (2x^{\star }- {z}^\star - x^{\star }- \gamma \nabla G(x^{\star }) ) {\Vert }) \\&= o({\Vert } {z}_{k}-{z}^\star {\Vert }) + O({|} \gamma _{k}-\gamma {|}) . \end{aligned} \end{aligned}$$

Now move **Term 1** to the other side of (35) and combine the definition of $\overline{R}$ and the Riemannian Taylor expansion [15, Lemma B.2], to obtain

$$\begin{aligned} \begin{aligned}&\gamma \tau _{k+1} \nabla _{\mathcal {M}_{x^{\star }}^{R}} R(u_{k+1}) \!-\! \gamma \nabla _{\mathcal {M}_{x^{\star }}^{R}} R(x^{\star }) - \left( {\tau _{k+1} \text {P}_{T^R_{u_{k+1}}} \!-\! \text {P}_{T^R_{x^{\star }}}}\right) (x^{\star }\!- {z}^\star \!- \gamma \nabla G(x^{\star }) ) \\&\quad = \text {P}_{T^R_{x^{\star }}}\nabla ^2_{\mathcal {M}_{x^{\star }}^{R}} \overline{R}(x^{\star }) \text {P}_{T^R_{x^{\star }}}(u_{k+1}-x^{\star }) + o({\Vert } {z}_{k}-x^{\star } {\Vert }) . \end{aligned} \end{aligned}$$

Owing to [15, Lemma 4.3], that the Riemannian Hessian $\text {P}_{T^R_{x^{\star }}}\nabla ^2_{\mathcal {M}_{x^{\star }}^{R}} \overline{R}(x^{\star }) \text {P}_{T^R_{x^{\star }}}$ is symmetric positive definite. For the term $\text {P}_{T^R_{x^{\star }}}(\gamma _{k}\nabla G(x_{k}) - \gamma \nabla G(x^{\star }))$, since we assume that *F* is locally $C^2$ around $x^{\star }$, we can apply the Taylor expansion:

$$\begin{aligned} \begin{aligned} {\gamma _{k}\nabla G(x_{k}) - \gamma \nabla G(x^{\star })}&=\gamma ( \nabla G(x_{k}) - \nabla G(x^{\star })) + (\gamma _{k}-\gamma )\nabla G(x_{k}) \\&= \text {P}_{V}\left( {\nabla F(x_{k}) - \nabla F(x^{\star })}\right) + O({|} \gamma _{k}-\gamma {|}) \\&= \text {P}_{V}\nabla ^2 F \text {P}_{V}( {z}_{k}-{z}^\star ) + o({\Vert } {z}_{k}-{z}^\star {\Vert }) + O({|} \gamma _{k}-\gamma {|}) . \end{aligned} \end{aligned}$$

Recall that ${H}_{\overline{R}}{:=} \text {P}_{T^R_{x^{\star }}}\nabla ^2_{\mathcal {M}_{x^{\star }}^{R}} \overline{R}(x^{\star }) \text {P}_{T^R_{x^{\star }}}$ and ${H}_{G}{:=} \text {P}_{V}\nabla ^2 F \text {P}_{V}$. Then, for (35),

36 $$\begin{aligned} \begin{aligned}&{H}_{\overline{R}}(u_{k+1}- x^{\star }) = 2\text {P}_{T^R_{x^{\star }}}(x_{k}- x^{\star }) - \text {P}_{T^R_{x^{\star }}}({z}_{k}- {z}^\star ) - \text {P}_{T^R_{x^{\star }}}(u_{k+1}- x^{\star }) \\&\qquad \qquad - \gamma {H}_{G}( {z}_{k}-{z}^\star ) + o({\Vert } {z}_{k}-{z}^\star {\Vert }) + O({|} \gamma _{k}-\gamma {|}) \\ \Longrightarrow \,&(\text {Id}+ {H}_{\overline{R}}) \text {P}_{T^R_{x^{\star }}}(u_{k+1}- x^{\star }) = 2\text {P}_{T^R_{x^{\star }}}(x_{k}- x^{\star }) - \text {P}_{T^R_{x^{\star }}}({z}_{k}- {z}^\star ) \\&\qquad \qquad \qquad - \gamma {H}_{G}( {z}_{k}-{z}^\star ) + o({\Vert } {z}_{k}-{z}^\star {\Vert }) + O({|} \gamma _{k}-\gamma {|}) \\ \Longrightarrow \,&\text {P}_{T^R_{x^{\star }}}(u_{k+1}- x^{\star }) = 2{M}_{\overline{R}}\text {P}_{V}({z}_{k}- {z}^\star ) - {M}_{\overline{R}}({z}_{k}-{z}^\star ) - \gamma {M}_{\overline{R}}{H}_{G}( {z}_{k}-{z}^\star ) \\&\qquad \qquad \qquad \qquad + o({\Vert } {z}_{k}-{z}^\star {\Vert }) + O({|} \gamma _{k}-\gamma {|}) \\ \Longrightarrow \,&u_{k+1}- x^{\star }= 2{M}_{\overline{R}}\text {P}_{V}({z}_{k}- {z}^\star ) - {M}_{\overline{R}}({z}_{k}-{z}^\star ) - \gamma {M}_{\overline{R}}{H}_{G}( {z}_{k}-{z}^\star ) \\&\qquad \qquad \qquad + o({\Vert } {z}_{k}-{z}^\star {\Vert }) + O({|} \gamma _{k}-\gamma {|}) , \end{aligned} \end{aligned}$$

where we used several times the relation $u_{k}-x^{\star }= \text {P}_{T^R_{x^{\star }}}(u_{k}-x^{\star }) + o({\Vert } u_{k}-x^{\star } {\Vert })$. Summing up (36) and $x_{k}- x^{\star }= \text {P}_{V} ({z}_{k}- {z}^\star )$ yields

$$\begin{aligned} \begin{aligned} ({z}_{k}+ u_{k+1}- x_{k}) - {z}^\star&= ({z}_{k}- {z}^\star ) + (u_{k+1}- x^{\star }) - (x_{k}- x^{\star }) \\&= \mathscr {M}_{\gamma }({z}_{k}- {z}^\star ) + o({\Vert } {z}_{k}-{z}^\star {\Vert }) + O({|} \gamma _{k}-\gamma {|}) . \end{aligned} \end{aligned}$$

Hence, for the non-stationary FDR iteration, we have

$$\begin{aligned} \begin{aligned} {z}_{k+1}-{z}^\star&= (1-\lambda _{k})({z}_{k}- {z}^\star ) + \lambda _{k}\left( { ({z}_{k}+ u_{k+1}- x_{k}) - ({z}^\star + x^{\star }- x^{\star }) }\right) \\&= (1-\lambda _{k})({z}_{k}- {z}^\star ) + \lambda _{k}\mathscr {M}_{\gamma }({z}_{k}- {z}^\star ) + o({\Vert } {z}_{k}-{z}^\star {\Vert }) + \chi _{k}\\&= \mathscr {M}_{\gamma ,\lambda }({z}_{k}- {z}^\star ) - (\lambda _{k}-\lambda )(\text {Id}-\mathscr {M}_{\gamma })({z}_{k}- {z}^\star ) + o({\Vert } {z}_{k}-{z}^\star {\Vert }) + \chi _{k}. \end{aligned} \end{aligned}$$

Since $\lim _{k\rightarrow +\infty } \tfrac{{\Vert } (\lambda _{k}-\lambda )(\text {Id}-\mathscr {M}_{\gamma })({z}_{k}- {z}^\star ) {\Vert }}{{\Vert } {z}_{k}-{z}^\star {\Vert }} \le \lim _{k\rightarrow +\infty } \tfrac{{|} \lambda _{k}-\lambda {|}{\Vert } \text {Id}-\mathscr {M}_{\gamma } {\Vert }{\Vert } {z}_{k}- {z}^\star {\Vert }}{{\Vert } {z}_{k}-{z}^\star {\Vert }} = 0$, then we get ${z}_{k+1}-{z}^\star = \mathscr {M}_{\gamma ,\lambda }({z}_{k}- {z}^\star ) + \psi _{k}+ \chi _{k}$ and conclude the proof. $\square$

Before presenting the local linear convergence result, we need to study the spectral properties of $\mathscr {M}_{\gamma ,\lambda }$, which is presented in the lemma below.

#### Lemma 5.2

Given $\gamma \in ]0, 2\beta _{{V}}[$ and $\lambda \in ]0, \frac{4\beta _{{V}}-\gamma }{2\beta _{{V}}}[$, we have that $\mathscr {M}_{\gamma }$ is $\frac{2\beta _{{V}}}{4\beta _{{V}}-\gamma }$-averaged and $\mathscr {M}_{\gamma ,\lambda }$ is $\frac{2\beta _{{V}}\lambda }{4\beta _{{V}}-\gamma }$-averaged. Moreover, for all *k* large enough

(i) $\mathscr {M}_{\gamma ,\lambda }$ converges to some matrix $\mathscr {M}_{\gamma }^{\infty }$ and,
  $$\begin{aligned} \mathscr {M}_{\gamma ,\lambda }^k - \mathscr {M}_{\gamma }^{\infty }= (\mathscr {M}_{\gamma ,\lambda }- \mathscr {M}_{\gamma }^{\infty })^k \ \text { and }\ \rho (\mathscr {M}_{\gamma ,\lambda }-\mathscr {M}_{\gamma }^{\infty }) < 1 . \end{aligned}$$
(ii) Given any $\rho \in ]\rho (\mathscr {M}_{\gamma ,\lambda }-\mathscr {M}_{\gamma }^{\infty }), 1[$, ${\Vert } \mathscr {M}_{\gamma ,\lambda }^k-\mathscr {M}_{\gamma }^{\infty } {\Vert } = O(\rho ^k)$.

#### Proof

Since ${W}_{\overline{R}}$ is firmly non-expansive by Lemma 5.1, it follows from [1, Example 4.7] that ${M}_{\overline{R}}$ is firmly non-expansive and hence  is non-expansive. Similarly, as $\text {P}_{V}$ is firmly non-expansive,  is non-expansive. As a result,  is firmly non-expansive [1, Proposition 4.21(i)–(ii)]. Then, given $\gamma \in [0, 2\beta _{{V}}]$, $\text {Id}- \gamma {H}_{G}$ is $\frac{2\beta _{{V}}}{4\beta _{{V}}-\gamma }$-averaged non-expansive. Therefore, owing to Lemma 4.1, we have the averaged property of $\mathscr {M}_{\gamma }$ and $\mathscr {M}_{\gamma ,\lambda }$. We deduce from [1, Proposition 5.15] that $\mathscr {M}_{\gamma }$ and $\mathscr {M}_{\gamma ,\lambda }$ are convergent, *i.e.* the limit of $\mathscr {M}_{\gamma ,\lambda }^k$ exists as *k* approaches $+\infty$. It is denoted as $\mathscr {M}_{\gamma }^{\infty }$. Moreover, $\mathscr {M}_{\gamma ,\lambda }^k - \mathscr {M}_{\gamma }^{\infty }= (\mathscr {M}_{\gamma ,\lambda }- \mathscr {M}_{\gamma }^{\infty })^k$, $\forall k \in \mathbb {N}$, and $\rho (\mathscr {M}_{\gamma ,\lambda }-\mathscr {M}_{\gamma }^{\infty }) < 1$ by [36, Theorem 2.12]. The second claim of the lemma is classical using the spectral radius formula; See *e.g.* [36, Theorem 2.12(i)]. $\square$

Owing to Lemma 5.2, we can further simplify the linearized iteration (34).

#### Corollary 5.1

Consider the non-stationary FDR iteration (6) and suppose that it is run under the assumptions of Theorem 5.2. Then the following holds:

(i) Iteration (34) is equivalent to
  37 $$\begin{aligned}&(\text {Id}-\mathscr {M}_{\gamma }^{\infty })({z}_{k+1}- {z}^\star ) \nonumber \\&\quad = (\mathscr {M}_{\gamma ,\lambda }-\mathscr {M}_{\gamma }^{\infty })(\text {Id}-\mathscr {M}_{\gamma }^{\infty }) ({z}_{k}- {z}^\star ) + (\text {Id}-\mathscr {M}_{\gamma }^{\infty }) \psi _{k}+ \chi _{k}. \end{aligned}$$
(ii) If moreover *R* is locally polyhedral around $x^{\star }$ and *F* is quadratic, then ${z}_{k+1}- {z}^\star = (\mathscr {M}_{\gamma ,\lambda }-\mathscr {M}_{\gamma }^{\infty })({z}_{k}- {z}^\star )$.

#### Proof

For the first claim. Let $K \in \mathbb {N}$ sufficiently large such that the locally linearized iteration (34) holds, then we have for all $k \ge K$38Since ${z}_{k}\rightarrow {z}^\star$ and $\mathscr {M}_{\gamma ,\lambda }$ is convergent to $\mathscr {M}_{\gamma }^{\infty }$ by Lemma 5.2, taking the limit as $k \rightarrow +\infty$, we have for all finite $p \ge K$,39Using (39) in (38), we get

$$\begin{aligned} \begin{aligned} {z}_{k+1}- {z}^\star&= (\mathscr {M}_{\gamma ,\lambda }- \mathscr {M}_{\gamma }^{\infty }) ({z}_{k}- {z}^\star ) + (\text {Id}-\mathscr {M}_{\gamma }^{\infty }) (\psi _{j} + \chi _{j}) + \mathscr {M}_{\gamma }^{\infty }({z}_{k+1}-{z}^\star ) . \end{aligned} \end{aligned}$$

It is also immediate to see from Lemma 5.2 that ${\Vert } \text {Id}-\mathscr {M}_{\gamma }^{\infty } {\Vert } \le 1$ and that $(\mathscr {M}_{\gamma ,\lambda }-\mathscr {M}_{\gamma }^{\infty })(\text {Id}-\mathscr {M}_{\gamma }^{\infty }) = \mathscr {M}_{\gamma ,\lambda }-\mathscr {M}_{\gamma }^{\infty }$. Rearranging the terms yields the claimed equivalence.

Under polyhedrality and constant parameters, we have from Theorem 5.2 that $o({\Vert } {z}_{k}-{z}^\star {\Vert })$ and $O(\lambda _{k}{|} \gamma _{k}-\gamma {|})$ vanish, and the result follows. $\square$

### Local Linear Convergence

We are now in position to claim local linear convergence of the FDR iterates.

#### Theorem 5.3

Consider the non-stationary FDR iteration (6) and suppose it is run under the conditions of Theorem 5.2. Let be $\rho \in ]\rho (\mathscr {M}_{\gamma ,\lambda }-\mathscr {M}_{\gamma }^{\infty }), 1[$ and $K\in \mathbb {N}$ such that, for all $k\ge K$, ${\Vert } \mathscr {M}_{\gamma ,\lambda }^k-\mathscr {M}_{\gamma }^{\infty } {\Vert } = O(\rho ^k)$ (see Lemma 5.2). Then the following holds:

(i) If there exists $\eta \in ]0, \rho [$ such that $\lambda _{k}{|} \gamma _{k}-\gamma {|} = O(\eta ^{k-K})$, then
  40 $$\begin{aligned} {\Vert } (\text {Id}-\mathscr {M}_{\gamma }^{\infty })({z}_{k}- {z}^\star ) {\Vert } = O(\rho ^{k-K}) . \end{aligned}$$
(ii) If moreover *R* is locally polyhedral around $x^{\star }$, *F* is quadratic, and that $(\gamma _{k}, \lambda _{k}) \equiv (\gamma ,\lambda ) \in ]0, 2\beta _{{V}}[ \times ]0, [$, then we have
  41 $$\begin{aligned} {\Vert } {z}_{k}- {z}^\star {\Vert } \le \rho ^{k-K} {\Vert } z_{K} - {z}^\star {\Vert } . \end{aligned}$$

#### Remark 5.3

- For the first case of Theorem 5.3, if $\mathscr {M}_{\gamma }^{\infty }= 0$ then we obtain the convergence rate directly on ${\Vert } {z}_{k}-{z}^\star {\Vert }$. Moreover, we can further derive the convergence rate of ${\Vert } x_{k}-x^{\star } {\Vert }$ and ${\Vert } u_{k}-x^{\star } {\Vert }$.
- The condition on $\lambda _{k}{|} \gamma _{k}-\gamma {|}$ in Theorem 5.3(i) implies that $\{\gamma _k\}_{k\in \mathbb {N}}$ should converge fast enough to $\gamma$. Otherwise, the local convergence rate would be dominated by that of $\lambda _{k}{|} \gamma _{k}-\gamma {|}$. Especially, if $\lambda _{k}{|} \gamma _{k}-\gamma {|}$ converges sublinearly to 0, then the local convergence rate will eventually become sublinear. See Fig. 2 in the experiments section for a numerical illustration.
- The above result can be easily extended to the case of GFB method, for the sake of simplicity we shall skip the details here. Nevertheless, numerical illustrations will be provided in Sect. 6.

#### Proof

For the first claim, let $K \in \mathbb {N}$ be sufficiently large such that (37) holds. We then have from Corollary 5.1(i)Since $\rho (\mathscr {M}_{\gamma ,\lambda }-\mathscr {M}_{\gamma }^{\infty }) < 1$ by Lemma 5.2, from the spectral radius formula, we know that for every $\rho \in ]\rho (\mathscr {M}_{\gamma ,\lambda }-\mathscr {M}_{\gamma }^{\infty }),1[$, there is a constant *C* such that ${\Vert } (\mathscr {M}_{\gamma ,\lambda }-\mathscr {M}_{\gamma }^{\infty })^j {\Vert } \le C \rho ^j$ holds for all integers *j*. We thus getBy assumption, $\chi _j = C'\eta ^j$ for some constant $C' \ge 0$ and $\eta <\rho$. Then we haveSetting $C^{''}=C( {\Vert } z_{K}-{z}^\star {\Vert } + \frac{ C' \eta ^{K} }{ \rho - {\eta }}) < +\infty$, we obtainThis, together with the fact that ${\Vert } (\text {Id}-\mathscr {M}_{\gamma }^{\infty })\psi _j {\Vert } = o({\Vert } (\text {Id}-\mathscr {M}_{\gamma }^{\infty })(z_j-{z}^\star ) {\Vert })$ yields the claimed result. The second claim follows from Corollary 5.1 that ${z}_{k}- {z}^\star = (\mathscr {M}_{\gamma ,\lambda }-\mathscr {M}_{\gamma }^{\infty })^{k+1-K}(z_{K} - {z}^\star )$ and we conclude the proof. $\square$

### Extension to Three-Operator Splitting

So far, we have presented the global and local convergence analysis of the FDR algorithm. As we recalled in the introduction, FDR is closely related with the three-operator splitting method (TOS) [7]. Therefore, it would be interesting to extend the obtained result to TOS. However, extending the global convergence result to TOS is far from straightforward. Hence, in the following, we mainly focus on the local aspect.

For the sake of notational simplicity, we rewrite problem (9) as

42 $$\begin{aligned} \min _{x\in \mathbb {R}^n} \left\{ \Psi (x) = F (x) + R(x) + J(x) \right\} , \end{aligned}$$

where we suppose the following assumptions:

1. $$J, R \in \Gamma _0(\mathbb {R}^{n})$$.
2. $F: \mathbb {R}^n \rightarrow \mathbb {R}$ is convex continuously differentiable with $\nabla F$ being $(1/\beta )$-Lipschitz continuous.
3. $\text {Argmin}(\Psi ) \ne \emptyset$, *i.e.* the set of minimizers is not empty.

Correspondingly, the TOS iteration (10) becomes

43 $$\begin{aligned} \begin{aligned} u_{k+1}&= \text {prox}_{\gamma R} \left( { 2x_{k}- {z}_{k}- \gamma \nabla F (x_{k}) }\right) \\ {z}_{k+1}&= {z}_{k}+ \lambda _{k}( u_{k+1}-x_{k}) , \\ x_{k+1}&= \text {prox}_{\gamma J}({z}_{k+1}) . \end{aligned} \end{aligned}$$

We suppose the following assumption on the algorithm parameters:

(B.4) The (constant) step size verifies $\gamma \in ]0, 2\beta [$ and the sequence of relaxation parameters $\{\lambda _{k}\}_{k\in \mathbb {N}}$ is such that $\sum _{k\in \mathbb {N}}\lambda _{k}(\frac{4\beta -\gamma }{2\beta }-\lambda _{k}) = +\infty$.

The fixed-point operator of TOS reads as

44 $$\begin{aligned} \mathscr {T}_{\gamma }= \text {Id}- \text {prox}_{\gamma R} + \text {prox}_{\gamma J}(2\text {prox}_{\gamma R} - \text {Id}- \gamma \nabla F \circ \text {prox}_{\gamma J}) , \end{aligned}$$

and $\mathscr {T}_{\gamma ,\lambda _{k}}= (1-\lambda _{k}) \text {Id}+ \lambda _{k}\mathscr {T}_{\gamma }$. Differently from $\mathscr {F}_{\gamma }$ (see (11)), $\mathscr {T}_{\gamma }$ cannot be simplified into a compact form.

#### Lemma 5.3

[7, Proposition 2.1 and Theorem 2.1] Consider the TOS iteration (43) and the fixed-point operator (44). Suppose that Assumptions (B.1)–(B.4) hold. Then,

(i) the operator $\mathscr {T}_{\gamma }$ is $\frac{2\beta }{4\beta -\gamma }$-averaged non-expansive.
(ii) $\{{z}_{k}\}_{k\in \mathbb {N}}$ converges to some ${z}^\star$ in $\text {fix}(\mathscr {T}_{\gamma })$; moreover, both $\{u_{k}\}_{k\in \mathbb {N}}$ and $\{x_{k}\}_{k\in \mathbb {N}}$ converge to $x^{\star }{:=} \text {prox}_{\gamma J}({z}^\star )$, which is a global minimizer of $\Psi$.

Similar to (30), under Lemma 5.3, we have the optimality condition

$$\begin{aligned} (x^{\star }- {z}^\star )/{\gamma } \in \nabla F (x^{\star }) + \partial R(x^{\star })\quad \text { and }\quad ({z}^\star - x^{\star })/{\gamma } \in \partial J (x^{\star }). \end{aligned}$$

Following Sects. 5.1–5.3, we present the local convergence of TOS.

*Finite Activity Identification* We start with the finite identification result, for both $u_{k}, x_{k}$ as *J* is no longer the indicator function of a subspace.

#### Corollary 5.2

For the TOS iteration (43). Suppose it is run under Assumptions (B.1)–(B.4), so that $(u_{k},x_{k},{z}_{k}) \rightarrow (x^{\star },x^{\star },{z}^\star )$ where ${z}^\star \in \text {fix}(\mathscr {T}_{\gamma })$ and $x^{\star }{:=} \text {prox}_{\gamma J}({z}^\star ) \in \text {Argmin}(\Psi )$. Moreover, suppose $R \in \text {PSF}_{x^{\star }}(\mathcal {M}_{x^{\star }}^{R})$, $J \in \text {PSF}_{x^{\star }}(\mathcal {M}_{x^{\star }}^{J})$, and the following non-degeneracy condition holds

45 $$\begin{aligned} (x^{\star }- {z}^\star )/{\gamma } - \nabla F (x^{\star }) \in \text {ri}\left( { \partial R(x^{\star }) }\right) \quad \text { and }\quad ({z}^\star - x^{\star })/{\gamma } \in \text {ri}\left( { \partial J (x^{\star }) }\right) . \end{aligned}$$

Then, there exists $K \in \mathbb {N}$ such that $(u_{k},x_{k}) \in \mathcal {M}_{x^{\star }}^{R}\times \mathcal {M}_{x^{\star }}^{J}$ for every $k \ge K$.

*Local Linearized Iteration* Define $\widetilde{R}(u) {:=} \gamma R(u) - \langle u,\,x^{\star }-{z}^\star -\gamma \nabla F(x^{\star }) \rangle$ and $\widetilde{J}(x) {:=} \gamma J(x) - \langle x,\,{z}^\star -x^{\star } \rangle$. We have the following corollary from Lemma 5.1.

#### Corollary 5.3

Suppose that $J \in \text {PSF}_{x^{\star }}(\mathcal {M}_{x^{\star }}^{J})$ and $R \in \text {PSF}_{x^{\star }}(\mathcal {M}_{x^{\star }}^{R})$. Then their Riemannian Hessians at $x^{\star }$ read

$$\begin{aligned} {H}_{\widetilde{J}}{:=} \text {P}_{T^J_{x^{\star }}}\nabla ^2_{\mathcal {M}_{x^{\star }}^{J}} \widetilde{J}(x^{\star }) \text {P}_{T^J_{x^{\star }}}\quad \text { and }\quad {H}_{\widetilde{R}}{:=} \text {P}_{T^R_{x^{\star }}}\nabla ^2_{\mathcal {M}_{x^{\star }}^{R}} \widetilde{R}(x^{\star }) \text {P}_{T^R_{x^{\star }}}, \end{aligned}$$

which are symmetric positive semi-definite under either of the two conditions:

(i) Condition (45) holds.
(ii) $\mathcal {M}_{x^{\star }}^{J}$ and $\mathcal {M}_{x^{\star }}^{R}$ are affine subspaces.

In turn, the matrices ${W}_{\widetilde{J}}{:=} (\text {Id}+{H}_{\widetilde{J}})^{-1} \text { and }{W}_{\widetilde{R}}{:=} (\text {Id}+{H}_{\widetilde{R}})^{-1}$ are both firmly non-expansive.

Now assume *F* is locally $C^2$-smooth around $x^{\star }$, and define ${H}_{F}{:=} \nabla ^2 F(x^{\star })$. Define also ${M}_{\widetilde{J}}{:=} \text {P}_{T^J_{x^{\star }}}{W}_{\widetilde{J}}\text {P}_{T^J_{x^{\star }}}$ and ${M}_{\widetilde{R}}{:=} \text {P}_{T^R_{x^{\star }}}{W}_{\widetilde{R}}\text {P}_{T^R_{x^{\star }}}$, and the matrices

$$\begin{aligned} \mathscr {L}_{\gamma }= \text {Id}+ 2{M}_{\widetilde{R}}{M}_{\widetilde{J}}- {M}_{\widetilde{R}}- {M}_{\widetilde{J}}- \gamma {M}_{\widetilde{R}}{H}_{F}{M}_{\widetilde{J}}\quad \text { and }\quad \mathscr {L}_{\gamma ,\lambda }= (1-\lambda ) \text {Id}+ \lambda \mathscr {L}_{\gamma }. \end{aligned}$$

#### Lemma 5.4

[7, Proposition 2.1] $\mathscr {L}_{\gamma }$ is $\frac{2\beta }{4\beta -\gamma }$-averaged non-expansive.

The above lemma entails that $\mathscr {L}_{\gamma }, \mathscr {L}_{\gamma ,\lambda }$ are convergent; hence, the spectral properties result in Lemma 5.2 applied to them. Denote $\mathscr {L}_{\gamma }^{\infty }{:=} \lim _{k\rightarrow +\infty } \mathscr {L}_{\gamma ,\lambda }^k$.

#### Corollary 5.4

Consider the TOS iteration (43). Suppose it is run under Assumptions (B.1)–(B.4), that $\lambda _{k}\rightarrow \lambda \in ]0, \frac{4\beta -\gamma }{2\beta }[$, and that *F* is locally $C^2$ around $x^{\star }$. Then we have ${z}_{k+1}- {z}^\star = \mathscr {L}_{\gamma ,\lambda }({z}_{k}- {z}^\star ) + o({\Vert } {z}_{k}- {z}^\star {\Vert })$. If moreover *J*, *R* are locally polyhedral around $x^{\star }$, *F* is quadratic and $\lambda _{k}\equiv \lambda \in ]0, \frac{4\beta -\gamma }{2\beta }[$ is chosen constant, then the term $o({\Vert } {z}_{k}- {z}^\star {\Vert })$ vanishes.

We can also specialize Corollary 5.1 to this context; however, we choose to skip it owing to its obviousness.

*Local Linear Convergence* Finally, we are able to present the local linear convergence for (43).

#### Corollary 5.5

For the TOS iteration (43). Suppose Assumptions (B.1)–(B.4) hold, and that $\lambda _{k}\rightarrow \lambda \in ]0, \frac{4\beta -\gamma }{2\beta }[$, and that *F* is locally $C^2$ around $x^{\star }$. Then

(i) Given any $\rho \in ]\rho (\mathscr {L}_{\gamma ,\lambda }-\mathscr {L}_{\gamma }^{\infty }), 1[$, there exists $K \in \mathbb {N}$ large enough such that ${\Vert } (\text {Id}-\mathscr {L}_{\gamma }^{\infty })({z}_{k}- {z}^\star ) {\Vert } = O(\rho ^{k-K}) \,\, \forall k \ge K$.
(ii) If moreover *J*, *R* are locally polyhedral around $x^{\star }$, *F* is quadratic and $\lambda _{k}\equiv \lambda \in ]0, \frac{4\beta -\gamma }{2\beta }[$ is chosen constant, then there exists $K \in \mathbb {N}$ such that ${\Vert } {z}_{k}- {z}^\star {\Vert } \le \rho ^{k-K} {\Vert } z_{K} - {z}^\star {\Vert }\,\,\forall k \ge K$.

## Numerical Experiments

In this section, we illustrate our theoretical results on problems arising from statistics, and signal/image processing applications.^3^

### Examples of Partly Smooth Functions

Table 1 provides some examples of popular partly smooth functions. More details about them can be found in [15, Sect. 5] and references therein.

**Table 1:** Examples of partly smooth functions

Function	Expression	Partial smooth manifold
$\ell _1$-norm	${\Vert } x {\Vert }_1=\sum _{i=1}^n {|} x_i {|}$	$\mathcal {M}= \big \{ z \in \mathbb {R}^n:\; I_z \subseteq I_x \big \} , I_x = \big \{ i:\; x_{i} \ne 0 \big \}$
$\ell _{1,2}$-norm		
$\ell _\infty$-norm	$\max _{i=\{1,\ldots ,n\}}{|} x_i {|}$	$\mathcal {M}= \big \{ z \in \mathbb {R}^n:\; z_{I_x} \in \mathbb {R}\text {sign}(x_{I_x}) \big \}$
TV semi-norm	${\Vert } x {\Vert }_{\mathrm{TV}}={\Vert } D_{\mathrm{DIF}}x {\Vert }_1$	$\mathcal {M}= \big \{ z \in \mathbb {R}^n:\; I_{D_{\mathrm{DIF}}z} \subseteq I_{D_{\mathrm{DIF}}x} \big \}$
Nuclear norm	${\Vert } x {\Vert }_*=\sum _{i=1}^r \sigma (x)$	$\mathcal {M}= \big \{ z \in \mathbb {R}^{n_1 \times n_2}:\; \text {rank}(z) = \text {rank}(x) = r \big \}$

For $x \in \mathbb {R}^n$ and some subset of indices ,  is the restriction of *x* to the entries indexed in . For $\ell _{\infty }$-norm, $I_x = \big \{ i:\; {|} x_i {|} = {\Vert } x {\Vert }_\infty \big \}$. $D_{\mathrm{DIF}}$ stands for the finite differences operator [37], $I_{D_{\mathrm{DIF}}x} = \{i: (D_{\mathrm{DIF}}x)_{i} \ne 0 \}$. $\text {sign}(x_{I_x})$ is the sign vector of $x_{I_x}$, and $\mathbb {R}\text {sign}(x_{I_x})$ is the span of $\text {sign}(x_{I_x})$. $\sigma (x)$ denotes the singular values of *x*

The $\ell _1$, $\ell _\infty$-norms and the anisotropic TV semi-norm are all polyhedral functions; hence, the corresponding Riemannian Hessians are simply 0. The $\ell _{1,2}$-norm is not polyhedral yet partly smooth relative to a subspace; the nuclear norm is partly smooth relative to the manifold of fixed-rank matrices, which is no longer a subspace. The Riemannian Hessian of these two functions is non-trivial and can be computed in the following [38].

### Numerical Experiments

*Global Convergence Rate of the Bregman Distance* We first demonstrate, numerically, the global *o*(1 / *k*) convergence rate of the Bregman divergence of Sect. 4. Towards this goal, we consider the fused LASSO problem [39]

46 $$\begin{aligned} \min _{x \in \mathbb {R}^n} \mu _1 {\Vert } x {\Vert }_{1} + \mu _{2} {\Vert } D_{\mathrm{DIF}}x {\Vert }_{1} + {\Vert } \mathcal {K}x-f {\Vert }^2 , \end{aligned}$$

where $\mu _1, \mu _2 > 0$ are trade-off weights. Note that all Assumptions (A.1)–(A.4) hold (in particular the set of minimizers is a non-empty compact set by coercivity of ${\Vert } \cdot {\Vert }_1$). The problem can be solved using the GFB instance of FDR in (8). In the test, we consider $n = 128$ and $\mathcal {K}\in \mathbb {R}^{36\times 128}$ is a random Gaussian matrix. The step size is chosen as $\gamma _{k} \equiv \frac{1}{4{\Vert } \mathcal {K} {\Vert }^2}$ such that we can observe the *o*(1 / *k*) convergence behaviour for enough number of iterations.

**Fig. 1:**
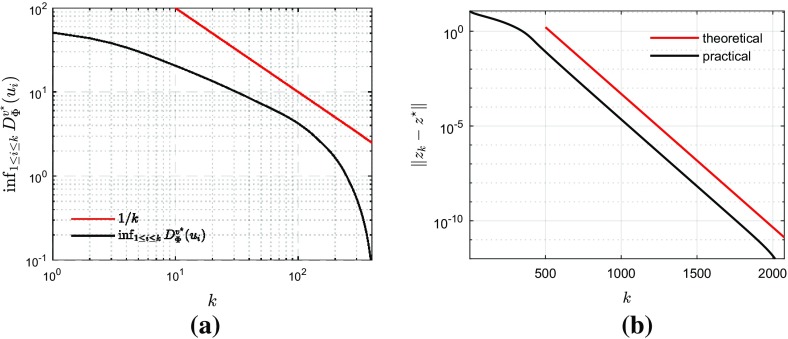
Results of applying (8) to solve the fused LASSO problem (46). **a** Convergence profile of the Bregman distance $\inf _{0 \le i \le k} \mathcal {D}_{{\Phi }}^{v^{\star }}\!(u_{i})$. **b** Convergence profile of ${\Vert } {z}_{k}-{z}^\star {\Vert }$

The convergence profile of $\min _{0 \le i \le k} \mathcal {D}_{{\Phi }}^{v^{\star }}\!(u_{i})$ is shown in Fig. 1a. The plot is in log-log scale, where the red line corresponds to the sublinear *O*(1 / *k*) rate and the black line is $\min _{0 \le i \le k} \mathcal {D}_{{\Phi }}^{v^{\star }}\!(u_{i})$. One can then confirm numerically the prediction of Theorem 4.2.

However, it can be observed that beyond some iteration, *e.g.*$10^2$ for the consider example, the convergence rate changes to linear. We argue in the next section that this is likely to be due to finite activity identification since $\ell _{1}$-norm and total variation are partly smooth (in fact even polyhedral) and that, for all *k* large enough, GFB enters into a local linear convergence regime.

*Local Linear Convergence of GFB/FDR* Following the above discussion, in Fig. 1b we present the local linear convergence of FDR in terms of ${\Vert } {z}_{k}-{z}^\star {\Vert }$ as we are in the scope of Theorem 5.3(ii). We use the same parameters setting as in Fig. 1a. The red line stands for the estimated rate (see Theorem 5.3), while the black line is numerical observation. The starting point of the red line is the number of iteration where $u_{k}$ identifies the manifolds. As shown in the figure, we indeed have local linear convergence behaviour of ${\Vert } {z}_{k}-{z}^\star {\Vert }$. Moreover, since $F = \frac{1}{2}{\Vert } \mathcal {K}x - f {\Vert }^2$ is quadratic, $\ell _{1}$-norm and total variation are polyhedral, our theoretical rate estimation is tight, *i.e.* the red line has the same slope as the black line.

*Non-stationary FDR* We now investigate the convergence behaviour of the non-stationary version of FDR and compare it to the stationary one. We fix $\lambda _k \equiv 1$, *i.e.* the iteration is unrelaxed. The stationary FDR algorithm is run with $\gamma = \beta$. For the non-stationary ones, four choices of $\gamma _k$ are considered:

$$\begin{aligned} \begin{aligned} \text {Case\,1:}&\, \gamma _k = \left( 1 + \tfrac{1}{k^{1.1}}\right) \beta ,\,\,&\text {Case\,2:}&\, \gamma _k = \left( 1 + \tfrac{1}{k^{2}}\right) \beta , \\ \text {Case\,3:}&\, \gamma _k = \left( 1 + 0.999^k\right) \beta ,\,\,&\text {Case\,4:}&\, \gamma _k = \left( 1 + 0.5^k\right) \beta . \end{aligned} \end{aligned}$$

**Fig. 2:**
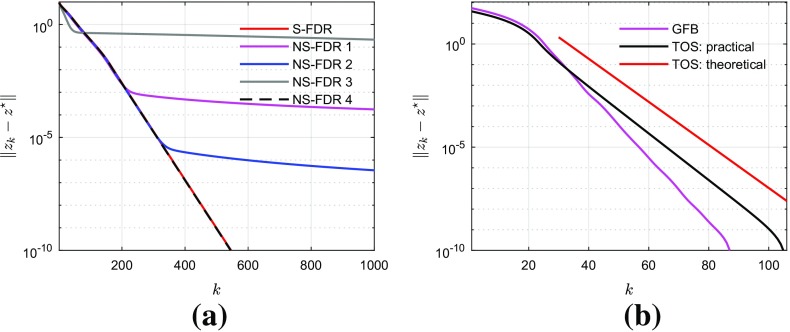
**a** Comparison of ${\Vert } {z}_{k}-{z}^\star {\Vert }$ between stationary (“S-FDR”) and non-stationary FDR (“NS-FDR X”, X stands for Case X). **b** Comparison of ${\Vert } {z}_{k}-{z}^\star {\Vert }$ between GFB and TOS for problem (47)

Obviously, we have $\gamma _{k}\rightarrow \gamma = \beta$ and $\sum _{k\in \mathbb {N}}{{|} \gamma _k - \gamma {|}} < +\infty$ for all cases. Problem (46) is considered. The comparison results are displayed in Fig. 2a.

We can make the following observations from the comparison:

- In agreement with our analysis, the local convergence behaviour of the non-stationary iteration is no better than the stationary one. This contrasts with the global behaviour where non-stationarity could be beneficial (see last comment hereafter);
- As argued in Remark 5.3(ii), the convergence rate is eventually controlled by the error ${|} \gamma _k-\gamma {|}$, except for “Case 4”, Indeed, 0.5 is strictly smaller than the local linear rate of the stationary version (*i.e.*${|} \gamma _k-\gamma {|} = o({\Vert } {z}_{k}-{z}^\star {\Vert })$);
- The non-stationary FDR seems to lead to faster identification, typically for “Case 3”. This is the effect of bigger step size at the early stage.

*Local Linear Convergence of GFB/TOS* To conclude the numerical experiments, we demonstrate the local convergence behaviour of GFB and TOS algorithms. Consider the non-negative low-rank matrix completion problem

47 $$\begin{aligned} \min _{x \in \mathbb {R}^{n \times n}} \mu {\Vert } x {\Vert }_{*} + \iota _{\mathbb {R}^{n \times n}_{+}}(x) + {\Vert } \mathcal {K}x-f {\Vert }^2 , \end{aligned}$$

where we recall that ${\Vert } \cdot {\Vert }_{*}$ is the nuclear norm (sum of singular values), and $\mathbb {R}^{n \times n}_+$ is the set of matrices with non-negative entries. Again, our main Assumptions (A.1)–(A.4) are verified thanks to continuity, convexity and coercivity. Problem (47) is a special instance of (42) if we let $F=\frac{1}{2}{\Vert } \mathcal {K}\cdot -f {\Vert }^2$, $R=\mu {\Vert } \cdot {\Vert }_{*}$ and $J=\iota _{\mathbb {R}^n_{+}}(x)$. Hence, it can be solved by the TOS scheme (43) and also by the GFB algorithm (8).

In the test, we consider $x \in \mathbb {R}^{50\times 50}$ and $\mathcal {K}$ is the subsampling operator (we did not consider larger problem size as computing the theoretical rate is very time and memory consuming). Figure 2b shows the convergence profiles of GFB/TOS. Similarly to the observation made in Fig. 1b, both GFB (magenta line) and TOS (black line) converge sublinearly from the beginning and eventually enter a linear convergence regime. The red line is our theoretical linear rate estimation of TOS. Moreover, for this example, the performances of two algorithms are very close, especially for the global sublinear regime.

## Perspectives and Open Problems

In this paper, we address the convergence properties of FDR algorithm from both global and local perspectives. The obtained results allow us to better understand the optimization problem (1) and FDR algorithm and moreover lay the foundation for our future research regarding several open problems.

The first open problem is the acceleration of FDR/GFB/TOS, or in general acceleration schemes for non-descent-type methods. In recent years, owing to the success of Nesterov’s optimal scheme [9] and FISTA [12], inertial technique has been widely adopted to speed up other non-descent-type operator splitting methods [40]. However, unlike the results in [9, 12], the acceleration effects of inertial technique for these non-descent-type methods are rather limited, or even slower than the original method [40, Chapter 4]. As a consequence, a proper acceleration scheme for non-descent methods, including FDR/GFB/TOS, with guaranteed acceleration remains an open problem.

Another direction for acceleration is the incremental version of these algorithms, particularly for GFB as the separable structure of $\sum _i R_i(x)$ in (7) is ideal for designing incremental schemes. Moreover, if *F* also has finite sum structure, *e.g.*$F(x) = \sum _{i=1}^{m} f_i(x)$, then similar to [41], we can consider incremental schemes for both smooth and non-smooth components of the problem.

The third perspective would be extending the obtained results to the non-Euclidean setting. More precisely, the proximal mapping of (3) is defined based on the Euclidean distance between *u* and *x*. By replacing the Euclidean distance with a Bregman distance, we obtain the Bregman-type splitting algorithms which are much more general. Generalizing the obtained results to Bregman-type splitting setting would be important and challenging.

For the local convergence analysis of FDR algorithm, we have to restrict ourselves to finite-dimensional Euclidean space, which is due to the fact that partial smoothness is only available in finite dimension. However, recently it is reported that finite identification also occurs for problems in infinite dimension, such as the off-the-grid compressive sensing [42]. As a result, proper extension of partial smoothness to the infinite dimension is required to explain these phenomena.

## Conclusions

In this paper, we studied global and local convergence properties of the Forward–Douglas–Rachford method. Globally, we established an *o*(1 / *k*) convergence rate of the best iterate and *O*(1 / *k*) ergodic rate in terms of a Bregman divergence criterion designed for the method. We also specialized the result to the case of Forward–Backward splitting method, for which we showed that the objective function of the method converges at an *o*(1 / *k*) rate. Then, locally, we proved the linear convergence of the sequence when the involved functions are moreover partly smooth. In particular, we demonstrated that the method identifies the active manifolds in finite time and that then it converges locally linearly at a rate that we characterized precisely. We also extended the local linear convergence result to the case of three-operator splitting method. Our numerical experiments supported the theoretical findings.

## Appendix: Riemannian Geometry

Let $\mathcal {M}$ be a $C^2$-smooth embedded submanifold of $\mathbb {R}^n$ around a point *x*. We denote, respectively, $\mathcal {T}_{\mathcal {M}}(x)$ and $\mathcal {N}_{\mathcal {M}}(x)$ the tangent and normal space of $\mathcal {M}$ at point near *x* in $\mathcal {M}$.

*Exponential Map* Geodesics generalize the concept of straight lines in $\mathbb {R}^n$, preserving the zero acceleration characteristic, to manifolds. Roughly speaking, a geodesic is locally the shortest path between two points on $\mathcal {M}$. We denote by $\mathfrak {g}(t;x, h)$ the value at $t \in \mathbb {R}$ of the geodesic starting at $\mathfrak {g}(0;x,h) = x \in \mathcal {M}$ with velocity $\dot{\mathfrak {g}}(t;x, h) = \tfrac{d\mathfrak {g}}{\mathrm{d}t}(t;x,h) = h \in \mathcal {T}_{\mathcal {M}}(x)$ (which is uniquely defined). For every $h \in \mathcal {T}_{\mathcal {M}}(x)$, there exists an interval *I* around 0 and a unique geodesic $\mathfrak {g}(t;x, h): I \rightarrow \mathcal {M}$ such that $\mathfrak {g}(0; x, h) = x$ and $\dot{\mathfrak {g}}(0;x, h) = h$. The mapping $\text {Exp}_x : \mathcal {T}_{\mathcal {M}}(x) \rightarrow \mathcal {M},\,\, h\mapsto \text {Exp}_{x}(h) = \mathfrak {g}(1;x, h)$ is called *Exponential map*.

*Parallel Translation* Given $x, x' \in \mathcal {M}$, let $\mathcal {T}_{\mathcal {M}}(x), \mathcal {T}_{\mathcal {M}}(x')$ be their corresponding tangent spaces. Define $\tau : \mathcal {T}_{\mathcal {M}}(x) \rightarrow \mathcal {T}_{\mathcal {M}}(x')$ the parallel translation along the unique geodesic joining *x* to $x'$, which is isomorphism and isometry w.r.t. the Riemannian metric.

*Riemannian Gradient and Hessian* For a vector $v \in \mathcal {N}_{\mathcal {M}}(x)$, the Weingarten map of $\mathcal {M}$ at *x* is the operator $\mathfrak {W}_{x}(\cdot , v): \mathcal {T}_{\mathcal {M}}(x) \rightarrow \mathcal {T}_{\mathcal {M}}(x)$ defined by $\mathfrak {W}_{x}(\cdot , v) = - \text {P}_{\mathcal {T}_{\mathcal {M}}(x)} \text {d} V[h]$ where *V* is any local extension of *v* to a normal vector field on $\mathcal {M}$. The definition is independent of the choice of the extension *V*, and $\mathfrak {W}_{x}(\cdot , v)$ is a symmetric linear operator which is closely tied to the second fundamental form of $\mathcal {M}$, see [43, Proposition II.2.1].

Let *J* be a real-valued function which is $C^2$ along the $\mathcal {M}$ around *x*. The covariant gradient of *J* at $x' \in \mathcal {M}$ is the vector $\nabla _{\mathcal {M}} J(x') \in \mathcal {T}_{\mathcal {M}}(x')$ defined by $\langle \nabla _{\mathcal {M}} J(x'),\,h \rangle = \tfrac{\mathrm{d}}{\mathrm{d}t} J\left( {\text {P}_{\mathcal {M}}(x'+th)}\right) \big |_{t=0} ,\,\, \forall h \in \mathcal {T}_{\mathcal {M}}(x')$, where $\text {P}_{\mathcal {M}}$ is the projection operator onto $\mathcal {M}$. The covariant Hessian of *J* at $x'$ is the symmetric linear mapping $\nabla ^2_{\mathcal {M}} J(x')$ from $\mathcal {T}_{\mathcal {M}}(x')$ to itself which is defined as $\langle \nabla ^2_{\mathcal {M}} J(x') h,\,h \rangle = \tfrac{\mathrm{d}^2}{\mathrm{d}t^2} J\left( {\text {P}_{\mathcal {M}}(x'+th)}\right) \big |_{t=0} ,\,\, \forall h \in \mathcal {T}_{\mathcal {M}}(x')$. This definition agrees with the usual definition using geodesics or connections [44]. Now assume that $\mathcal {M}$ is a Riemannian embedded submanifold of $\mathbb {R}^{n}$, and that a function *J* has a $C^2$-smooth restriction on $\mathcal {M}$. This can be characterized by the existence of a $C^2$-smooth extension (representative) of *J*, *i.e.* a $C^2$-smooth function $\widetilde{J}$ on $\mathbb {R}^{n}$ such that $\widetilde{J}$ agrees with *J* on $\mathcal {M}$. Thus, the Riemannian gradient $\nabla _{\mathcal {M}}J(x')$ is given by $\nabla _{\mathcal {M}} J(x') = \text {P}_{\mathcal {T}_{\mathcal {M}}(x')} \nabla \widetilde{J}(x')$ and $\forall h \in \mathcal {T}_{\mathcal {M}}(x')$, the Riemannian Hessian reads $\nabla ^2_{\mathcal {M}} J(x') h = \text {P}_{\mathcal {T}_{\mathcal {M}}(x')} \nabla ^2 \widetilde{J}(x') h + \mathfrak {W}_{x'}\left( {h, \text {P}_{\mathcal {N}_{\mathcal {M}}(x')}\nabla \widetilde{J}(x')}\right)$, where the last equality comes from [45, Theorem 1]. When $\mathcal {M}$ is an affine or linear subspace of $\mathbb {R}^{n}$, then obviously $\mathcal {M}= x + \mathcal {T}_{\mathcal {M}}(x)$, and $\mathfrak {W}_{x'}(h, \text {P}_{\mathcal {N}_{\mathcal {M}}(x')} \nabla \widetilde{J}(x')) = 0$, and we have $\nabla ^2_{\mathcal {M}} J(x') = \text {P}_{\mathcal {T}_{\mathcal {M}}(x')} \nabla ^2 \widetilde{J}(x') \text {P}_{\mathcal {T}_{\mathcal {M}}(x')}$.

## Footnotes

1 For the interplay between the error bound property, the Kurdyka–Łojasiewicz property, and the quadratic growth property; see [20, 21].

2 It is symmetric, if and only if *R* is a non-degenerate convex quadratic form.

3 MATLAB source for reproducing the numerical result can be found at https://github.com/jliang993/Rate-FDR.
